# Magnetic dipolar interaction between correlated triplets created by singlet fission in tetracene crystals

**DOI:** 10.1038/ncomms9602

**Published:** 2015-10-12

**Authors:** Rui Wang, Chunfeng Zhang, Bo Zhang, Yunlong Liu, Xiaoyong Wang, Min Xiao

**Affiliations:** 1 National Laboratory of Solid State Microstructures, School of Physics, Nanjing University, Nanjing 210093, China; 2 Synergetic Innovation Center in Quantum Information and Quantum Physics, University of Science and Technology of China, Hefei, Anhui 230026, China; 3 Collaborative Innovation Center of Advanced Microstructures, Nanjing University, Nanjing 210093, China; 4 Department of Physics, University of Arkansas, Fayetteville, Arkansas 72701, USA

## Abstract

Singlet fission can potentially break the Shockley–Queisser efficiency limit in single-junction solar cells by splitting one photoexcited singlet exciton (S_1_) into two triplets (2T_1_) in organic semiconductors. A dark multiexciton state has been proposed as the intermediate connecting S_1_ to 2T_1_. However, the exact nature of this multiexciton state, especially how the doubly excited triplets interact, remains elusive. Here we report a quantitative study on the magnetic dipolar interaction between singlet-fission-induced correlated triplets in tetracene crystals by monitoring quantum beats relevant to the multiexciton sublevels at room temperature. The resonances of multiexciton sublevels approached by tuning an external magnetic field are observed to be avoided, which agrees well with the theoretical predictions considering a magnetic dipolar interaction of ∼0.008 GHz. Our work quantifies the magnetic dipolar interaction in certain organic materials and marks an important step towards understanding the underlying physics of the multiexciton state in singlet fission.

## Abstract (web summary)

The exact mechanism of singlet fission remains unresolved. Here, Wang *et al*. report a quantitative measurement of the interaction between singlet-fission-induced correlated triplets in tetracene crystals with quantum beat spectroscopy, indicating the role played by exciton delocalization in singlet fission.

## Graphical abstract

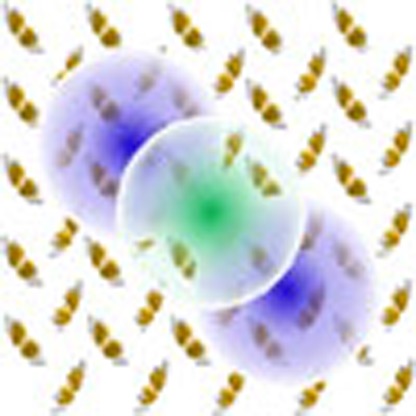

The spin-allowed singlet fission (SF) process is highly efficient in some organic semiconductors^1^,^2^,^3^,^4^,^5^,^6^,^7^,^8^,^9^. By creating two triplet excitons from a single photoexcited singlet exciton, the SF process of carrier multiplication can be implanted in multiple device architectures to improve the efficiency of solar conversion^10^,^11^,^12^,^13^,^14^. However, the intrinsic mechanism responsible for the fast fission process is still under intense debate, despite remarkable progresses have recently been made^2^,^3^,^4^,^5^,^6^,^7^,^8^,^15^,^16^,^17^,^18^,^19^,^20^,^21^,^22^. The essential role played by the intermediate multiexciton (ME) state (^1^(TT)) has been realized^1^,^2^,^3^,^4^,^5^,^15^,^17^,^20^ with the SF process being described as^1^,


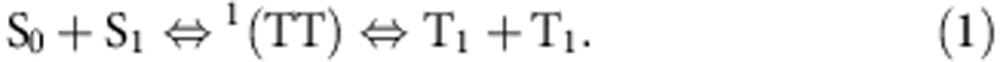


The doubly excited ME state, also referred to as the correlated pair of triplet excitons, which are entangled with spin coherence, is created when a singlet-excited molecule shares its energy with a neighbouring molecule at its ground state. The magnetic interaction between the correlated triplets is insightful for revealing the nature of the ME state by providing valuable information about their spatial separation and the effect of spin coherence^17^,^20^,^23^. However, it is challenging to quantify the weak magnetic dipolar interaction between the correlated triplet excitons at the ME state with a short lifetime^18^,^20^.

Magnetic dipolar interaction is pivotal for many technically significant processes in organic materials including exciton fission/fusion^1^, organic magneto-resistance^24^ and organic photovoltaics^25^. In a SF sensitizer, a conceivable approach to quantify the magnetic dipolar interaction is to investigate the interaction-induced energy shift of the ME state. In crystalline tetracene, the energy differences between the ME sublevels can be monitored through quantum beating signals in the singlet population^17^,^20^,^26^. As depicted in Fig. 1a, exciton fusion from two ME sublevels induces quantum beats in population of the S_1_ state, manifesting themselves as an oscillation in the time-resolved fluorescence (TRFL) spectrum. After first introduced in 1980s^26^, this quantum beat phenomenon in tetracene has recently been re-examined and comprehensively explained by Bardeen's group^17^,^20^,^27^. Being a direct evidence of the ME state, the quantum beating signal has been regarded as a fingerprint of SF in tetracene^15^,^17^,^20^. However, the signature of magnetic dipolar interaction has never been directly captured in tetracene^20^,^26^, and only an upper bound of ∼0.2 GHz was given due to the limitations in previous experiment^20^.

Here we exploit a new scenario of the interaction-induced anti-crossing to quantify the interaction strength in crystalline tetracene. We manipulate the ME sublevels by applying an external magnetic field to approach the level-crossing resonance. The magnetic dipolar interaction between the correlated triplets causes an avoided level crossing, resulting in two branches of levels with an energy gap. The interaction strength is proportional to the gap size that can be precisely evaluated by measuring the frequencies of quantum beats relevant to the resultant upper and lower levels with TRFL spectroscopy. With this approach, we have succeeded in quantitatively determining the magnetic dipolar interaction between the correlated triplet excitons at the ME state to be ∼0.008 GHz. The unexpected weak interaction strength indicates that it is necessary to consider the exciton size for a comprehensive understanding of the SF process.

## Results

### Theoretical consideration

**Figure 1:**
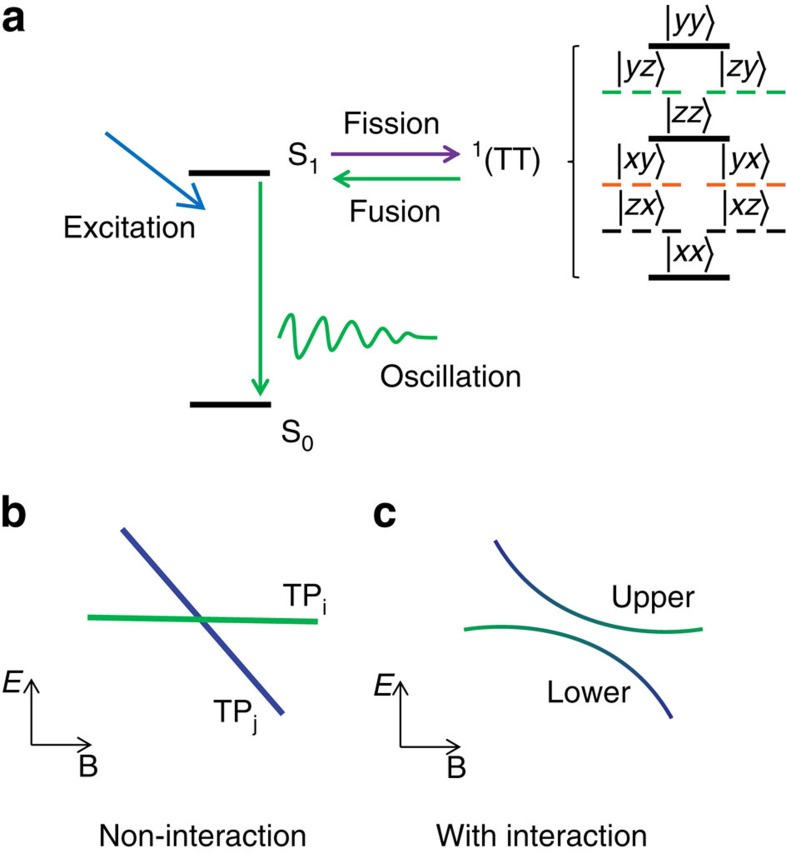
Interaction-induced anti-crossing among ME sublevels. (**a**) The scenario of the quantum beats related to the ME sublevels in tetracene crystals at zero field. Exciton fusion from sublevels of ME state induces the delayed fluorescence where quantum beats manifest themselves as oscillations in the TRFL spectrum. (**b**,**c**) The field dependences of two near-resonant sublevels for the cases of non-interaction and with interaction between correlated triplets, respectively. With magnetic interaction, the emergence of mixed states results in an avoided crossing with separated upper and lower branches (**c**).

Figure 1 schematically shows the scenario of the quantum beats relevant to the ME sublevels involving in the SF process. We describe the sublevels of ME state with a spin-dependent Hamiltonian of two correlated triplets. The Hamiltonian includes two isolated triplets (for example, *α* and *β*) and their mutual interaction (*H*_int_)^20^, that is,


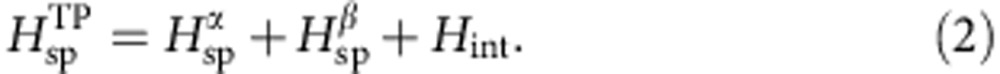


The isolated triplet dipole (for example, *α* or *β*, as represented by ‘Tri') can be described by the Hamiltonian as^20^,^28^


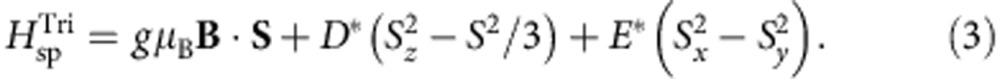


Here the first item represents the Zeeman shift due to the applied magnetic field, where **S**, **B**, *μ*_*B*_ and *g* are the spin operator, the external magnetic field, the Bohr magneton and the Lande *g*-factor, respectively. The latter two terms give zero-field Hamiltonian with parameters of D* and E* characterized by ESR experiments^29^. The Hamiltonian for the magnetic dipolar interaction between the correlated triplet dipoles can be written as^24^


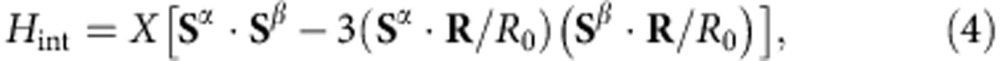


where **S**^*α*^ and **S**^*β*^ are the spin operators of the two triplets, **R** is the displacement vector between the two triplets with the magnitude of *R*_0_ and *X* is the parameter of interaction strength. As described in Supplementary Note 1, we calculate the energies of the ME sublevels by solving the Hamiltonian of equation (2) with a basis of nine eigenstates 
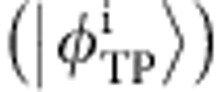
. In spite of weak magnitude, the interaction part can cause substantial differences from the energy levels predicted by the interaction-free model (Supplementary Figs 1 and 2). Those sublevels with non-zero mapping to the S_1_ state 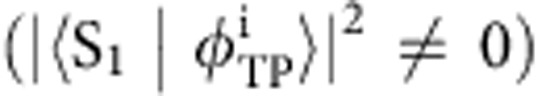 involve in the exciton fission/fusion processes and directly contribute to the quantum beats (Fig. 1a)^20^.

**Figure 2:**
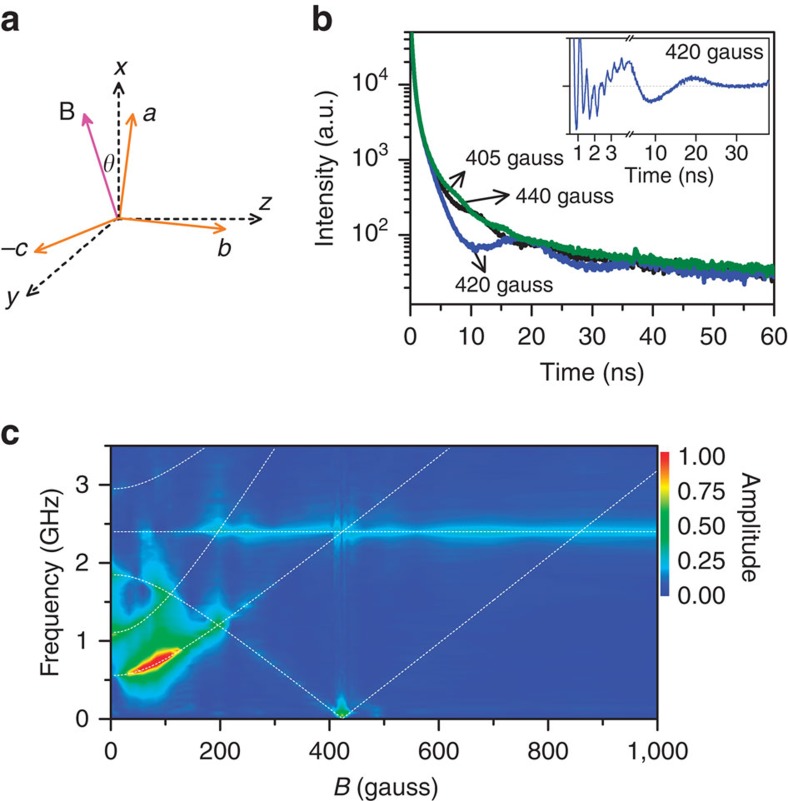
Magnetic-field-dependent quantum beats. (**a**) Schematic diagram of the external magnetic field applied at the *xy* plane with a small angle (*θ*) tilted relative to the *x* axis. (**b**) The decay curves of fluorescence dynamics with three different external magnetic field value tilted at *θ*=2° near the resonance of level crossing. Inset shows the oscillation part obtained by subtracting the multi-exponential decay components from the raw data recorded at 420 G. (**c**) The relative amplitudes of quantum beats are plotted as functions of the beat frequency and the field magnitude. The dashed lines indicate the expected beat frequencies calculated with interaction-free model.

Theoretical calculation predicts a sublevel crossing with a magnetic field of ∼420 G applied along the *x* axis (Supplementary Fig. 1). The mutual interaction opens a gap of Δ≈2*X* at the level-crossing resonance (Fig. 1c, Supplementary Fig. 1b,d). Since the gap size is small, it is difficult to directly measure the beat frequencies in time domain due to damping of the oscillations^20^. To overcome this obstacle, we slightly tilt the external magnetic field with a small angle (*θ*) relative to the *x* axis in the *xy* plane (Fig. 2a) to introduce an extra perturbation (*δE*_y_) in the Hamiltonian. The gap size then increases to be Δ≈2*X*+2*δE*_y_ (Supplementary Note 2), so the evaluation becomes more accurate because the visibility of quantum beat is significantly enhanced in the TRFL trace.

### Level-crossing resonance

Figure 2b plots the TRFL traces recorded under different magnetic fields at *θ*∼2°. An anomalous oscillation with the frequency of ∼0.05 GHz emerges due to the newly opened gap when the field is at 420 G (inset, Fig. 2b). The field-dependent amplitudes and frequencies of the measured quantum beats (Fig. 2c) are compared with theoretical calculations (Supplementary Fig. 3) to further confirm the above assignments. The interaction-free model can well explain the observed high-frequency beats (>0.1 GHz) (Supplementary Fig. 3), but fails to account for the anomalous part (∼420 G) without considering the opened energy gap (Δ). A control experiment has been performed with the magnetic field applied at *z* direction (Supplementary Note 3). Under this configuration, the anomalous beat no longer exists since no level crossing is found (Supplementary Figs 6 and 7), verifying that the observed low-frequency quantum beat is indeed a result of gap opened by the magnetic dipolar interaction and perturbation.

**Figure 3:**
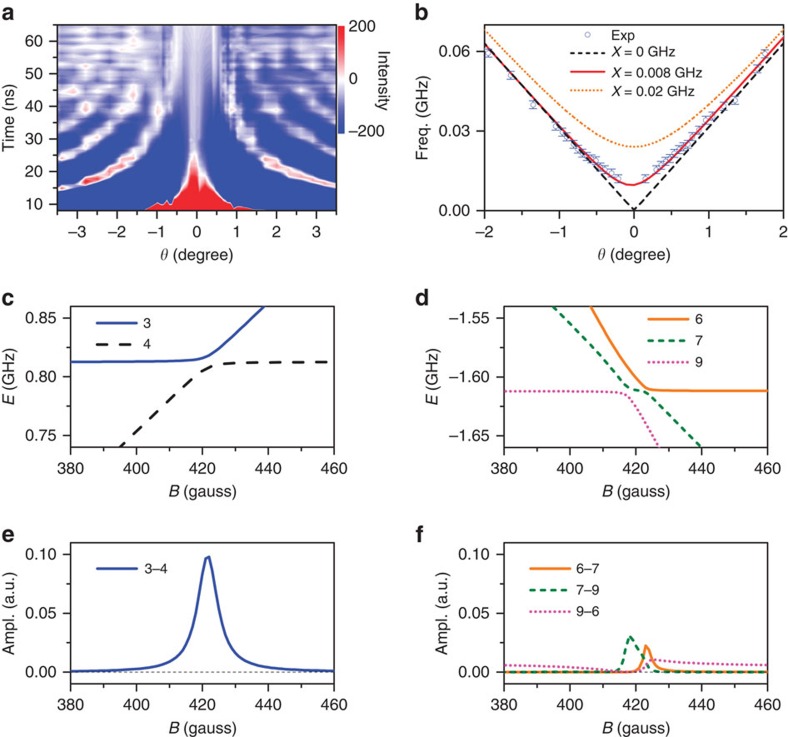
Quantum beats near level-crossing resonances. (**a**) The oscillation components are plotted versus the delay time and tilt angle (*θ*). The external field is set at 420 G. (**b**) Experimental results of *θ*-dependent beat frequencies are compared with theoretical calculated curves considering different strengths (*X*) of magnetic dipolar interaction. (**c**–**f**) Theoretical considerations of the interaction-induced anti-crossing of various ME sublevels (for details, see Supplementary Note 1). At the resonance field of ∼420 G, the anti-crossing occurs in two cases, resulting in the mixed states of three and four sublevels (**c**) and the mixed states of sixth, seventh, and ninth sublevels (**d**). The field-dependent amplitudes of low-frequency quantum beats calculated approximately as the mapping products of selected sublevel pairs for these two cases are plotted in **e** and **f**, respectively.

Next, we analyse the *θ*-dependent oscillatory components to quantify the interaction strength (Fig. 3a). We assume the displacement vector (**R**/*R*_0_) to be along the nearest neighbour orientation^3^,^16^. The resonant energies of all nine ME sublevels are calculated by exactly diagonalizing Hamiltonian (2) and indexed by their zero-field energies. When the magnetic dipolar interaction is included (Supplementary Fig. 1), the avoided crossing occurs between the three and four sublevels (Fig. 3c) as well as the sixth, seventh and ninth sublevels (Fig. 3d) at ∼420 G. The mapping products of 
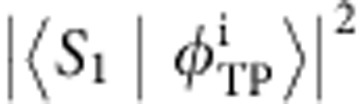
 for selected sublevel pairs, which directly reflect the beating amplitudes, are compared in Fig. 3e,f, suggesting the dominant contribution from the third and fourth sublevels (Supplementary Note 2; Supplementary Fig. 5). In Fig. 3b, the measured peak oscillation frequency of this anomalous beating signal as a function of the tilt angle *θ* is compared with the theoretical curves of energy separation between three and four sublevels calculated with different interaction strengths. The perturbation induced by field tilting corresponds to the zero-interaction curve (*X*=0) in Fig. 3b with *δE*_y_∝*θ* (Supplementary Fig. 4). The disparity of experimental data from the linear dependence on the tilt angle near *θ*≈0 is an evidence of existing magnetic dipolar interaction, which can be best reproduced by calculation with the interaction strength of *X*=0.008 GHz (Fig. 3b).

### Strong magnetic field limit

**Figure 4:**
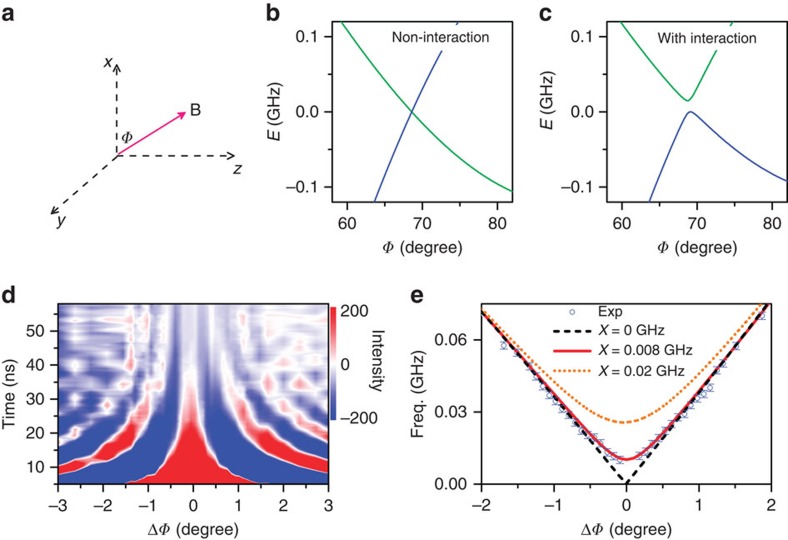
Quantum beats at the strong magnetic field limit. (**a**) Schematic diagram of a strong field applied at the *xz* plane having an angle *Φ* with respect to the *x* axis. In this strong field limit, only two dominant sublevels are involved in the fission and fusion processes. (**b**,**c**) The calculated energy alignments of two sublevels for the cases of non-interaction and with interaction between correlated triplets, respectively. The level crossing occurs at *Φ*≈69°. (**d**) The oscillation components are plotted versus the delay time and the angle (Δ*Φ*) deviated from the resonant value. The magnitude of the external field is ∼3,000 G. (**e**) The experimentally measured beating frequencies are plotted as a function of Δ*Φ* under the strong field limit. The curves are theoretically calculated results with different strengths of magnetic interaction.

Furthermore, we have also examined the quantum beats at the strong magnetic field limit. A field of ∼3,000 G is applied in the *xz* plane with an angle of Φ to the *x* axis as shown in Fig. 4a. In this regime, multiple beat frequencies collapse into one frequency, because only two sublevels dominate the mappings to the S_1_ state (Supplementary Fig. 2; Supplementary Note 4)^27^. The energy alignments of the two sublevels calculated using models with and without the interaction are shown in Fig. 4c,b, respectively. The two sublevels approach to degeneracy at Φ≈69° (Fig. 4b), which is avoided due to the magnetic dipolar interaction (Fig. 4c). The oscillatory amplitude is plotted as a function of delay time and the angle deviated from the degeneracy value (ΔΦ) in Fig. 4d. The measured ΔΦ-dependent beat frequencies shown in Fig. 4e agree well with the calculated curve having *X*=0.008 GHz. The value agrees well with that obtained at 420 G, which is significantly larger than the bounds of errors and spurious effects including the hyperfine coupling as discussed in Supplementary Methods (Supplementary Table 3). The strength measured here is at the same order of magnitude as that adopted to interpret the data of optically detected magnetic resonance of germinate triplet pair at the T_1_+T_1_ state in bis(triisopropylsilylethynyl)-tetracene films^23^. The weak magnitude measured here is also in consistence with the upper bound estimated in literature^20^ and theoretical analysis of other magnetic effects^30^. It is over two orders of magnitude smaller than the energy difference between the sublevels at zero field, which might explain why the signature of this magnetic dipolar interaction was absent in previous studies^20^,^26^.

## Discussion

The orientation of displacement vector (**R**/*R*_0_) with respect to the *z* axis of magnetic tensor may affect the evaluation of the interaction strength. We have considered all four possible configurations for two neighbouring molecules in *ab* plane (Supplementary Fig. 8). The interaction strength *X* that best reproduces the experimental data (Figs 3f and 4d) is far below the theoretical values for two nearest neighbour molecules (Supplementary Tables 1 and 2). The separation distance between two triplets at the ME state equivalent to the measured value of *X* can be estimated to be ∼1.7 nm, which is about two to four times of the intermolecular distance (Supplementary Note 5). The unexpected large separation between the correlated triplets can be plausibly caused by the delocalization effect of photoexcited singlet excitons^31^. Previous PL study has suggested the size of singlet excitons to be in the order of tens of molecules at low temperature^31^, which might also affect the exciton dynamics in certain extent at room temperature^7^. The pair of triplets resulted from the delocalized singlet excitons can distribute over a distance of multiple molecules, which can naturally explain the measured weak magnetic interaction.

Moreover, though the diffusion of triplet excitons at the ME state remains poorly understood, triplet diffusion at the T_1_+T_1_ state has been shown to be quite efficient in tetracene crystals^32^. In principle, the triplet diffusion may increase the separation distance between the triplet excitons over time and cause a time-dependent *X*. Such effect has not been clearly seen in this study, which could be ascribed as the consequence of quantum beat spectroscopy used in this study. As depicted in Fig. 1a, only the correlated triplet pairs that recombine through the exciton fusion process contribute significantly to the beating signal. The triplet pairs that diffuse apart may lose the spin coherence and/or the coherence needed for exciton fusion. Those triplet pairs generate delayed fluorescence but do not make significant contribution to the beating signal. Consequently, the time dependence of interaction strength is not manifested in the quantum beat spectroscopy here. Additional work will be needed to fully uncover the effect of exciton diffusion on the interaction between the triplet excitons at the ME state.

Overall, the spins of correlated triplets are weakly coupled with the environment, making our method reliable in evaluating the magnetic dipolar interaction at room temperature. This work represents the first successful quantification of the magnetic dipolar interaction between the correlated triplet excitons at the ME state in crystalline tetracene. The weak interaction strength observed here provides a direct evidence that the initial step (S_0_+S_1_↔^1^(TT)) of SF process involves molecules that are not nearest to each other. The entangled triplets at the ME state delocalized over many molecules arising from the delocalization of initial singlet excitons could be a plausible origin of the weak interaction strength. Such phenomena indicate that an alternative scenario different from the available models considering two proximately coupled molecules may be necessary to fully understand the SF process in crystalline tetracene. Including the effect of exciton delocalization to elucidate SF may be enlightening to address the debates between a charge-transfer mechanism and coherent superposition model responsible for the generation of intermediate ME states^2^,^16^,^19^,^20^,^33^,^34^,^35^. The quantum beating behaviour in crystalline tetracene due to spin coherence of the correlated triplet pairs is sensitive to the magnitude and direction of the magnetic field. These features deserve more in-depth investigations and can stimulate further adventures in applying SF to many new areas of research such as spintronics and quantum information science^36^,^37^,^38^.

## Methods

### Sample preparation and magnetic field alignment

Tetracene single crystals with the thickness of ∼1 μm and size up to 5 × 5 mm^2^ were prepared by the method of physical vapour deposition. The crystallographic axes were determined by X-ray diffraction and polarization microscopy (Supplementary Fig. 9), which were further employed to determine the magnetic axes with a transform matrix as established by electron spin resonance measurements (Supplementary Methods). A rotatable magnetic coil was used for magnetic-field-dependent experiments. The samples were mounted on a multi-axis platform to realize the desired alignment with respect to the magnetic field.

### Optical characterizations

The second harmonic field (400 nm) of the pulses emitted from a Ti:Sapphire oscillator (800 nm, Vitara, Coherent) was chosen as the excitation source. The repetition rate was reduced down to 4 MHz by a pulse picker and the excitation flux was kept at a low level (∼4 nJ cm^−2^) to avoid the effect of exciton–exciton annihilation. The emission light was collected by a fibre and routed to a spectrograph. The TRFL spectra at 530 nm were recorded with the technique of time-correlated single-photon counting by an avalanche photodiode having a temporal resolution of better than 50 ps. The multi-exponential decay components were subtracted from TRFL traces before Fourier transform to extract the frequencies and amplitudes of quantum beating signals (Supplementary Fig. 10).

### Theoretical simulation

The resonant energies of ME sublevels are calculated by exact diagonalization of the full spin-dependent Hamiltonian. Quantum beat in the detected TRFL signal is modelled by the dynamics of the density matrix elements governed by the quantum Liouville equations (Supplementary Note 1).

## Additional information

**How to cite this article:** Wang, R. *et al*. Magnetic dipolar interaction between correlated triplets created by singlet fission in tetracene crystals. *Nat. Commun.* 6:8602 doi: 10.1038/ncomms9602 (2015).

## Supplementary Material

Supplementary Information Supplementary Figures 1-10, Supplementary Tables 1-3, Supplementary Notes 1-5, Supplementary Methods and Supplementary References.

## References

[b1] Smith M. B. & Michl J. Recent advances in singlet fission. Ann. Rev. Phys. Chem. 64, 361–386 (2013). 23298243 10.1146/annurev-physchem-040412-110130

[b2] Chan W.-L., Ligges M. & Zhu X. Y. The energy barrier in singlet fission can be overcome through coherent coupling and entropic gain. Nat. Chem. 4, 840–845 (2012). 23000998 10.1038/nchem.1436

[b3] Zimmerman P. M., Bell F., Casanova D. & Head-Gordon M. Mechanism for singlet fission in pentacene and tetracene: From single exciton to two triplets. J. Am. Chem. Soc. 133, 19944–19952 (2011). 22084927 10.1021/ja208431r

[b4] Chan W.-L. . Observing the multiexciton state in singlet fission and ensuing ultrafast multielectron transfer. Science 334, 1541–1545 (2011). 22174249 10.1126/science.1213986

[b5] Zimmerman P. M., Zhang Z. & Musgrave C. B. Singlet fission in pentacene through multi-exciton quantum states. Nat. Chem. 2, 648–652 (2010). 20651727 10.1038/nchem.694

[b6] Grumstrup E. M., Johnson J. C. & Damrauer N. H. Enhanced triplet formation in polycrystalline tetracene films by femtosecond optical-pulse shaping. Phys. Rev. Lett. 105, 257403 (2010). 21231627 10.1103/PhysRevLett.105.257403

[b7] Burdett J. J., Mueller A. M., Gosztola D. & Bardeen C. J. Excited state dynamics in solid and monomeric tetracene: The roles of superradiance and exciton fission. J. Chem. Phys. 133, 144506 (2010). 20950016 10.1063/1.3495764

[b8] Thorsmolle V. K. . Morphology effectively controls singlet-triplet exciton relaxation and charge transport in organic semiconductors. Phys. Rev. Lett. 102, 017401 (2009). 19257238 10.1103/PhysRevLett.102.017401

[b9] Smith M. B. & Michl J. Singlet fission. Chem. Rev. 110, 6891–6936 (2010). 21053979 10.1021/cr1002613

[b10] Thompson N. J. . Energy harvesting of non-emissive triplet excitons in tetracene by emissive pbs nanocrystals. Nat. Mater. 13, 1039–1043 (2014). 25282507 10.1038/nmat4097

[b11] Tabachnyk M. . Resonant energy transfer of triplet excitons from pentacene to pbse nanocrystals. Nat. Mater. 13, 1033–1038 (2014). 25282509 10.1038/nmat4093

[b12] Tritsch J. R., Chan W.-L., Wu X., Monahan N. R. & Zhu X. Y. Harvesting singlet fission for solar energy conversion via triplet energy transfer. Nat. Commun. 4, 2679 (2013). 24145737 10.1038/ncomms3679

[b13] Congreve D. N. . External quantum efficiency above 100% in a singlet-exciton-fission-based organic photovoltaic cell. Science 340, 334–337 (2013). 23599489 10.1126/science.1232994

[b14] Hanna M. C. & Nozik A. J. Solar conversion efficiency of photovoltaic and photoelectrolysis cells with carrier multiplication absorbers. J. Appl. Phys. 100, 074510 (2006).

[b15] Zhang B. . Nonlinear density dependence of singlet fission rate in tetracene films. J. Phys. Chem. Lett. 5, 3462–3467 (2014). 26278594 10.1021/jz501736y

[b16] Yost S. R. . A transferable model for singlet-fission kinetics. Nat. Chem. 6, 492–497 (2014). 24848234 10.1038/nchem.1945

[b17] Piland G. B., Burdett J. J., Dillon R. J. & Burdett J. J. Singlet fission: From coherences to kinetics. J. Phys. Chem. Lett. 5, 2312–2319 (2014). 26279552 10.1021/jz500676c

[b18] Wilson M. W. B. . Temperature-independent singlet exciton fission in tetracene. J. Am. Chem. Soc. 135, 16680–16688 (2013). 24148017 10.1021/ja408854u

[b19] Beljonne D., Yamagata H., Bredas J. L., Spano F. C. & Olivier Y. Charge-transfer excitations steer the davydov splitting and mediate singlet exciton fission in pentacene. Phys. Rev. Lett. 110, 226402 (2013). 23767738 10.1103/PhysRevLett.110.226402

[b20] Burdett J. J. & Bardeen C. J. Quantum beats in crystalline tetracene delayed fluorescence due to triplet pair coherences produced by direct singlet fission. J. Am. Chem. Soc. 134, 8597–8607 (2012). 22530591 10.1021/ja301683w

[b21] Marciniak H. . Ultrafast exciton relaxation in microcrystalline pentacene films. Phys. Rev. Lett. 99, 176402 (2007). 17995352 10.1103/PhysRevLett.99.176402

[b22] Liu Y. . Large optical nonlinearity induced by singlet fission in pentacene films. Angew. Chem. Int. Ed. Engl. 54, 6222–6226 (2015). 25845461 10.1002/anie.201501396

[b23] Bayliss S. L. . Geminate and nongeminate recombination of triplet excitons formed by singlet fission. Phys. Rev. Lett. 112, 238701 (2014). 24972236 10.1103/PhysRevLett.112.238701

[b24] Wagemans W. . Spin-spin interactions in organic magnetoresistance probed by angle-dependent measurements. Phys. Rev. Lett. 106, 196802 (2011). 21668186 10.1103/PhysRevLett.106.196802

[b25] Zhang Y. . Spin-enhanced organic bulk heterojunction photovoltaic solar cells. Nat. Commun. 3, 1043 (2012). 22948825 10.1038/ncomms2057

[b26] Chabr M., Wild U. P., Funfschilling J. & Zschokkegranacher I. Quantum beats of prompt fluorescence in tetracene crystals. Chem. Phys. 57, 425–430 (1981).

[b27] Burdett J. J., Piland G. B. & Bardeen C. J. Magnetic field effects and the role of spin states in singlet fission. Chem. Phys. Lett. 585, 1–10 (2013).

[b28] Benk H. & Sixl H. Theory of 2 coupled triplet-states application to bicarbene structures. Mol. Phys. 42, 779–801 (1981).

[b29] Yarmus L., Rosentha J. & Chopp M. Epr of triplet excitons in tetracene crystals-spin polarization and role of singlet exciton fission. Chem. Phys. Lett. 16, 477–481 (1972).

[b30] Piland G. B., Burdett J. J., Kurunthu D. & Bardeen C. J. Magnetic field effects on singlet fission and fluorescence decay dynamics in amorphous rubrene. J. Phys. Chem. C 117, 1224–1236 (2013).

[b31] Lim S. H., Bjorklund T. G., Spano F. C. & Bardeen C. J. Exciton delocalization and superradiance in tetracene thin films and nanoaggregates. Phys. Rev. Lett. 92, 107402 (2004). 15089241 10.1103/PhysRevLett.92.107402

[b32] Akselrod G. M. . Visualization of exciton transport in ordered and disordered molecular solids. Nat. Commun. 5, 3646 (2014). 24736470 10.1038/ncomms4646

[b33] Busby E. . A design strategy for intramolecular singlet fission mediated by charge-transfer states in donor–acceptor organic materials. Nat. Mater. 14, 426–433 (2015). 25581625 10.1038/nmat4175

[b34] Aryanpour K., Shukla A. & Mzaumdar S. Theory of singlet fission in polyacenes, acene crystals, and covalently linked acene dimers. J. Phys. Chem. C 119, 6966–6979 (2015).

[b35] Kolomeisky A. B., Feng X. & Krylov A. I. A simple kinetic model for singlet fission: A role of electronic and entropic contributions to macroscopic rates. J. Phys. Chem. C 118, 5188–5195 (2014).

[b36] Grinolds M. S. . Nanoscale magnetic imaging of a single electron spin under ambient conditions. Nat. Phys. 9, 215–219 (2013).

[b37] Wilson A. C. . Tunable spin-spin interactions and entanglement of ions in separate potential wells. Nature 512, 57–60 (2014). 25100480 10.1038/nature13565

[b38] Kotler S., Akerman N., Navon N., Glickman Y. & Ozeri R. Measurement of the magnetic interaction between two bound electrons of two separate ions. Nature 510, 376–380 (2014). 24943952 10.1038/nature13403

