# Supplementary material for: Magnetic dipolar interaction between correlated triplets created by singlet fission in tetracene crystals
Source: Nat Commun. 2015 Oct 12;6:8602. doi: 10.1038/ncomms9602 (PMC4633952; doi:10.1038/ncomms9602)
Supplement: Supplementary Information — Supplementary Figures 1-10, Supplementary Tables 1-3, Supplementary Notes 1-5, Supplementary Methods and Supplementary References. [file ncomms9602-s1.pdf]

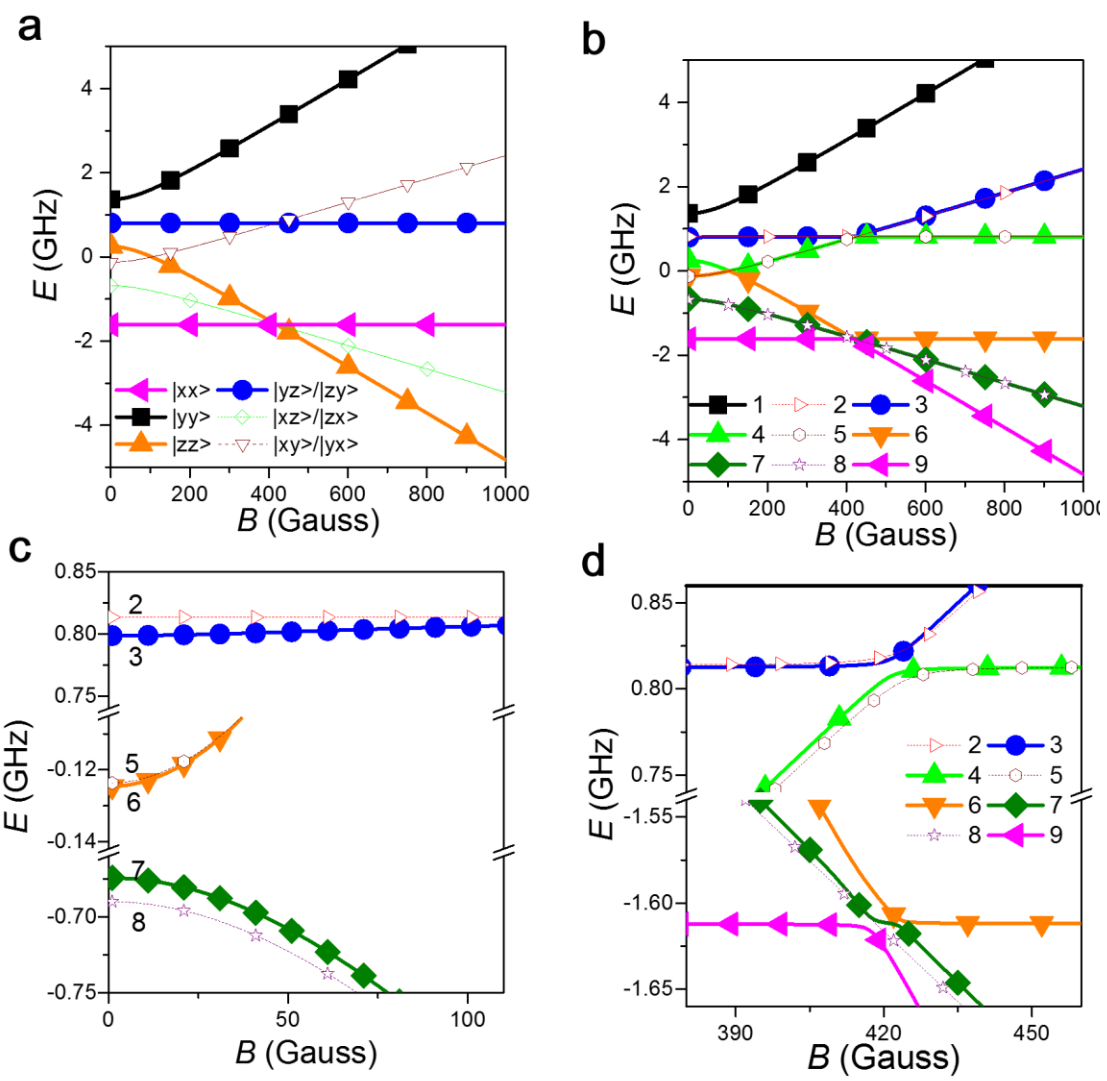

Supplementary Figure 1| Magnetic-field-dependent energies of the ME sublevels. The magnetic field is applied along x axis. The calculated eigenenergies of nine ME sublevels are plotted as a function of field magnitude. The results shown in **a** and **b** are calculated with the interaction-free and interaction-involved models, respectively. The levels with zero and non-zero mappings to the singlet state are represented by solid lines (solid symbols) and dashed lines (open symbols), respectively. The ME sublevels for interaction-involved model are sorted by their resonant energies at zero field. **c** and **d** are the same as **b** plotted in a magnified scale for clarity with different magnetic field ranges.

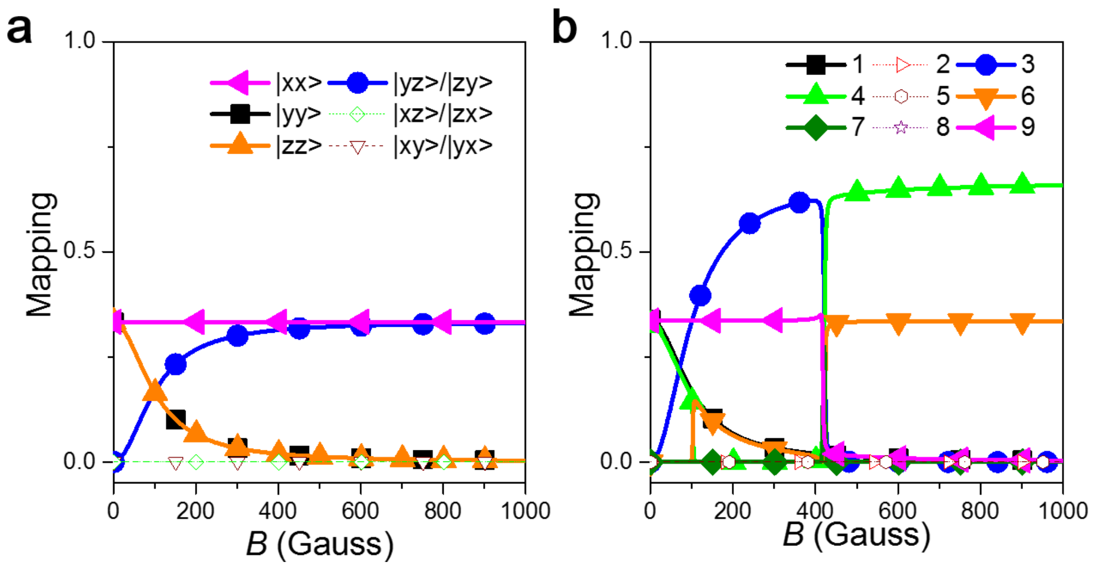

Supplementary Figure 2| Mappings of ME sublevels to the singlet state S_1. a and **b** are calculated results of the mappings of ME sublevels to the singlet state with the interaction-free and interaction-involved models, respectively. The ME sublevels are defined in the same way as in Supplementary Fig. 1.

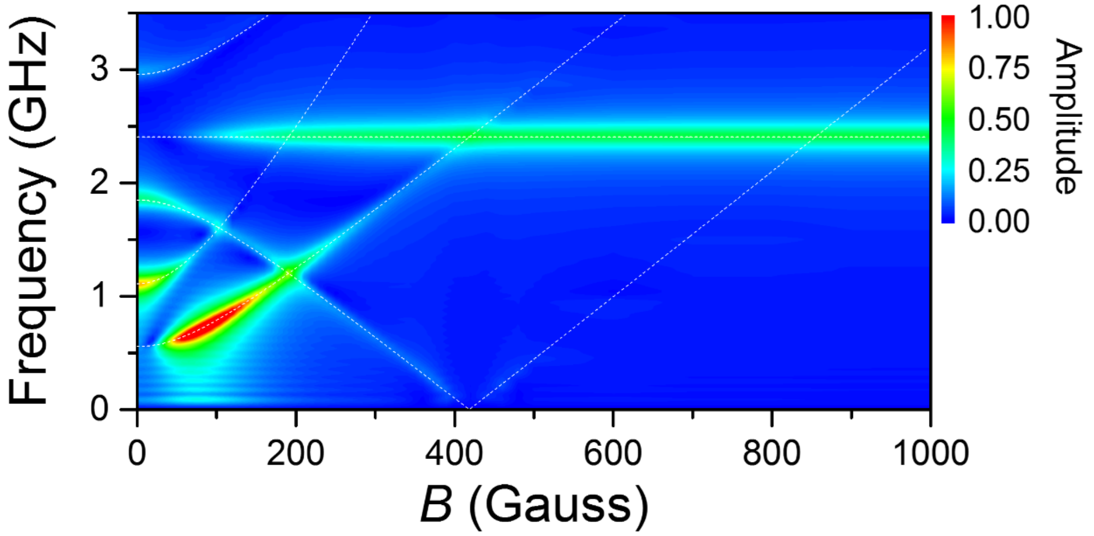

Supplementary Figure 3| Simulations of quantum beats with magnetic field along x axis. The calculated amplitudes of quantum beating are plotted as functions of the beat frequency and field magnitude. The dashed lines indicate the beat frequencies calculated under the interaction-free condition (Supplementary Fig. 1a).

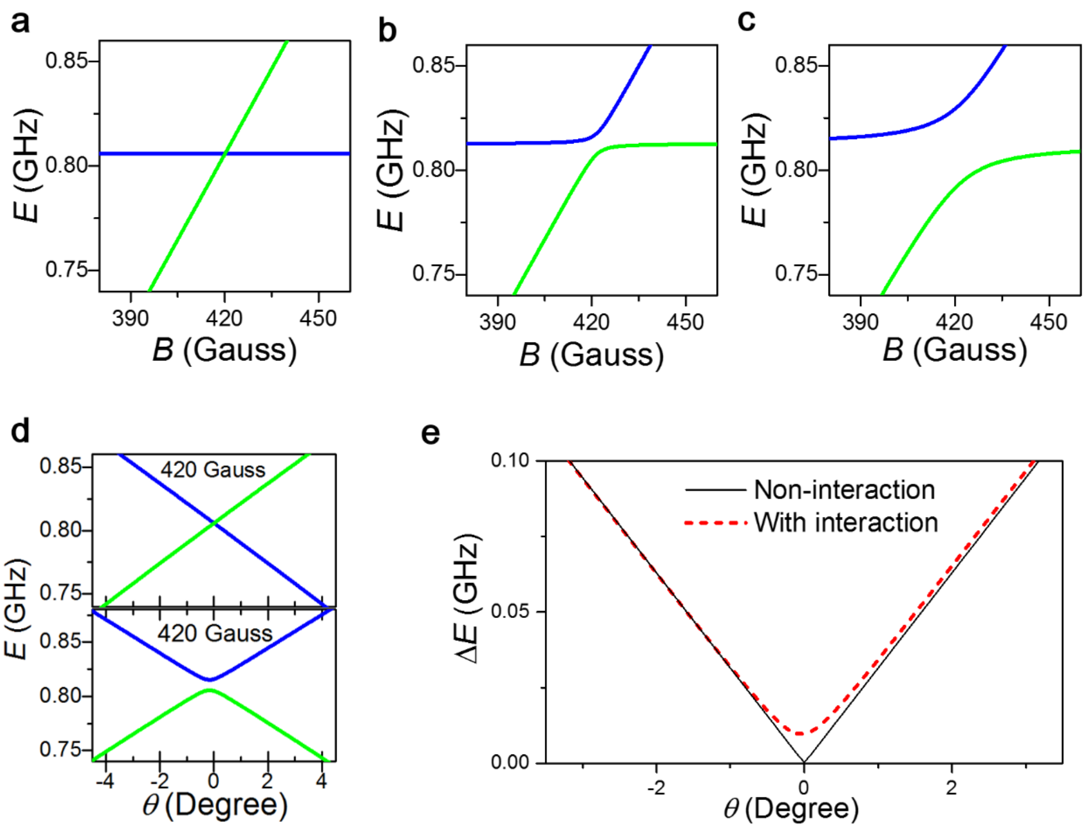

Supplementary Figure 4| Energies of ME sublevels (3 & 4) near level-crossing resonance. The magnetic field is applied along x axis. The results are calculated with the interaction-free model (**a**), the interaction-involved model (**b**), and the interaction-involved model with field tilted at 1.5 degrees (**c**). **d**, The energies of 3 & 4 sublevels with field of ~ 420 Gauss are plotted as a function of the tilt angle (θ). The data are calculated with the interaction-free (upper) and interaction-involved models (lower), respectively. **e**, The tilt-angle-dependent gap size is calculated with the interaction-free and interaction-involved models, respectively.

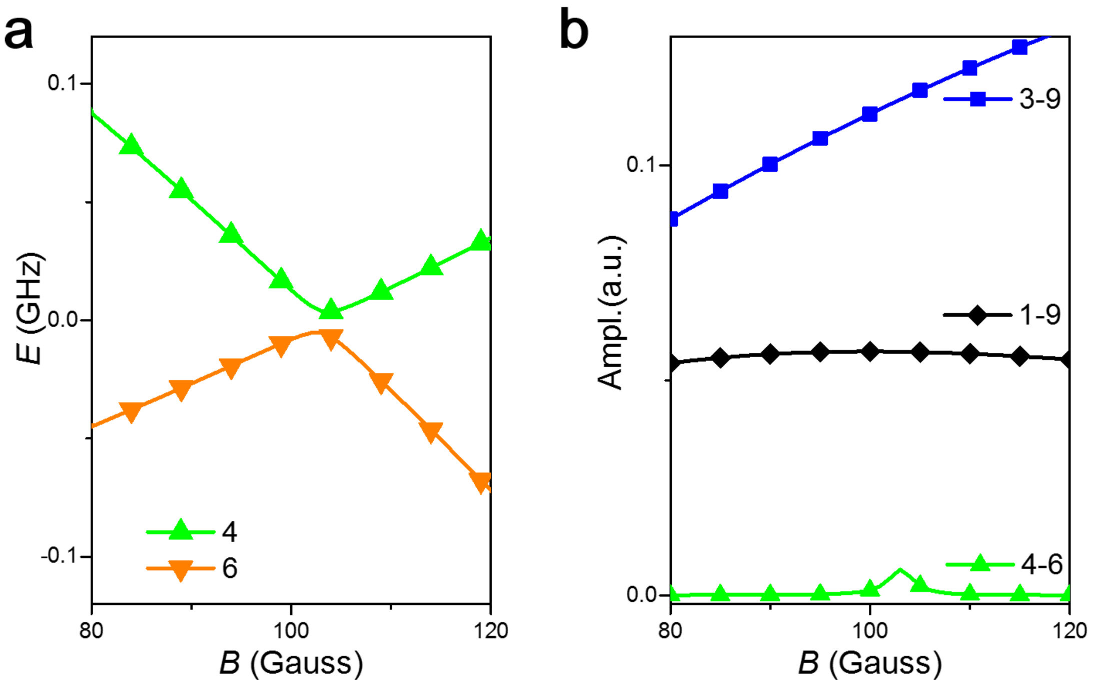

Supplementary Figure 5| Interaction-induced anti-crossing of ME sublevels of 4 and 6. a, Energies of ME sublevels of 4 and 6 near the level crossing resonance calculated with the interaction-involved model. **b**, The calculated beating amplitude related to ME sublevels 4 & 6 is compared with those related to sublevels of 1 & 9 and sublevels 3 & 9, respectively.

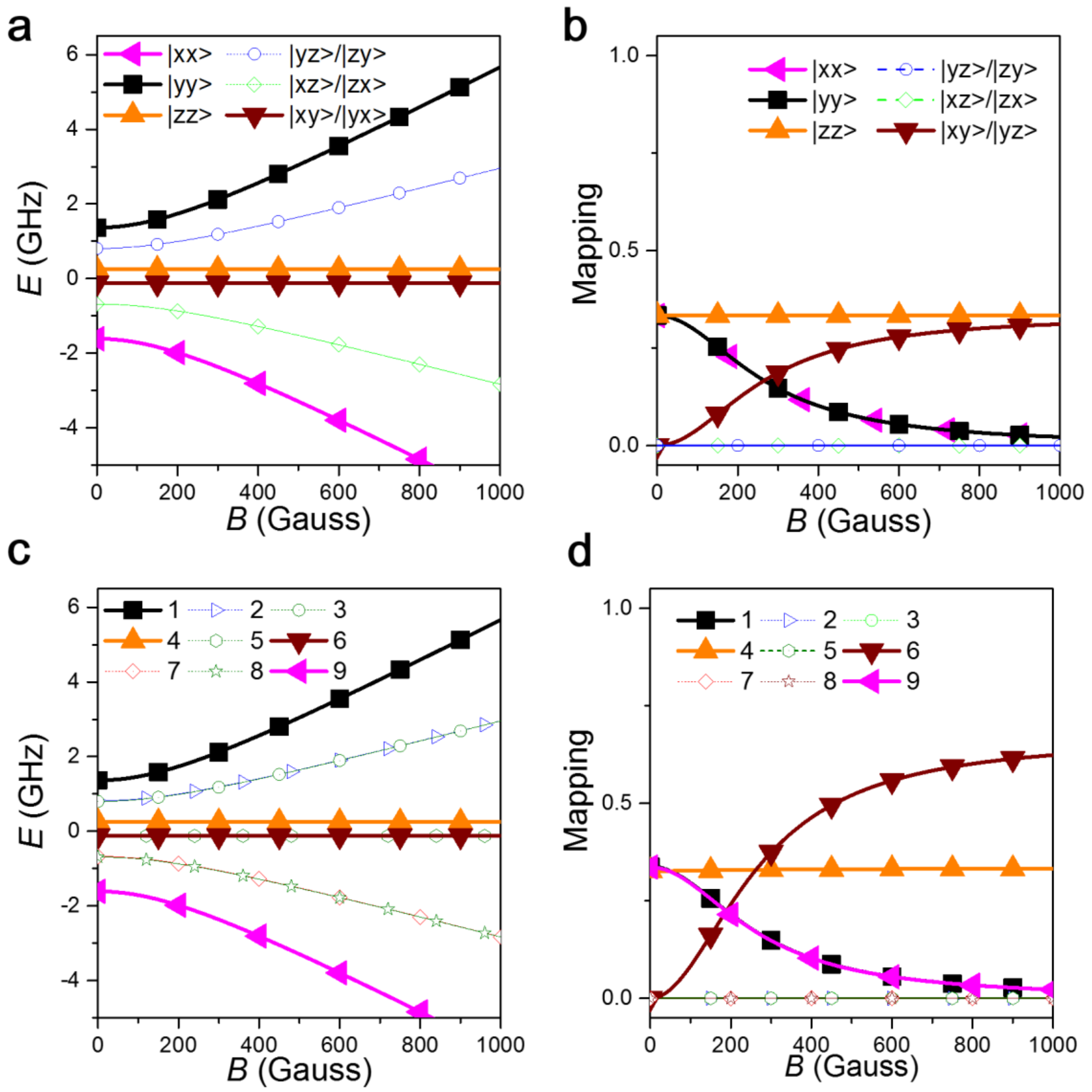

Supplementary Figure 6| Magnetic-field-dependent ME sublevels with field along z axis. With the interaction-free model, the calculated field-dependent energies and the mappings of the ME sublevels to the singlet state are plotted in (**a**) and (**b**), respectively. The calculation considering the magnetic dipolar interaction predicts the field-dependent energies and the mappings of the ME sublevels to the singlet state in (**c**) and (**d**), respectively.

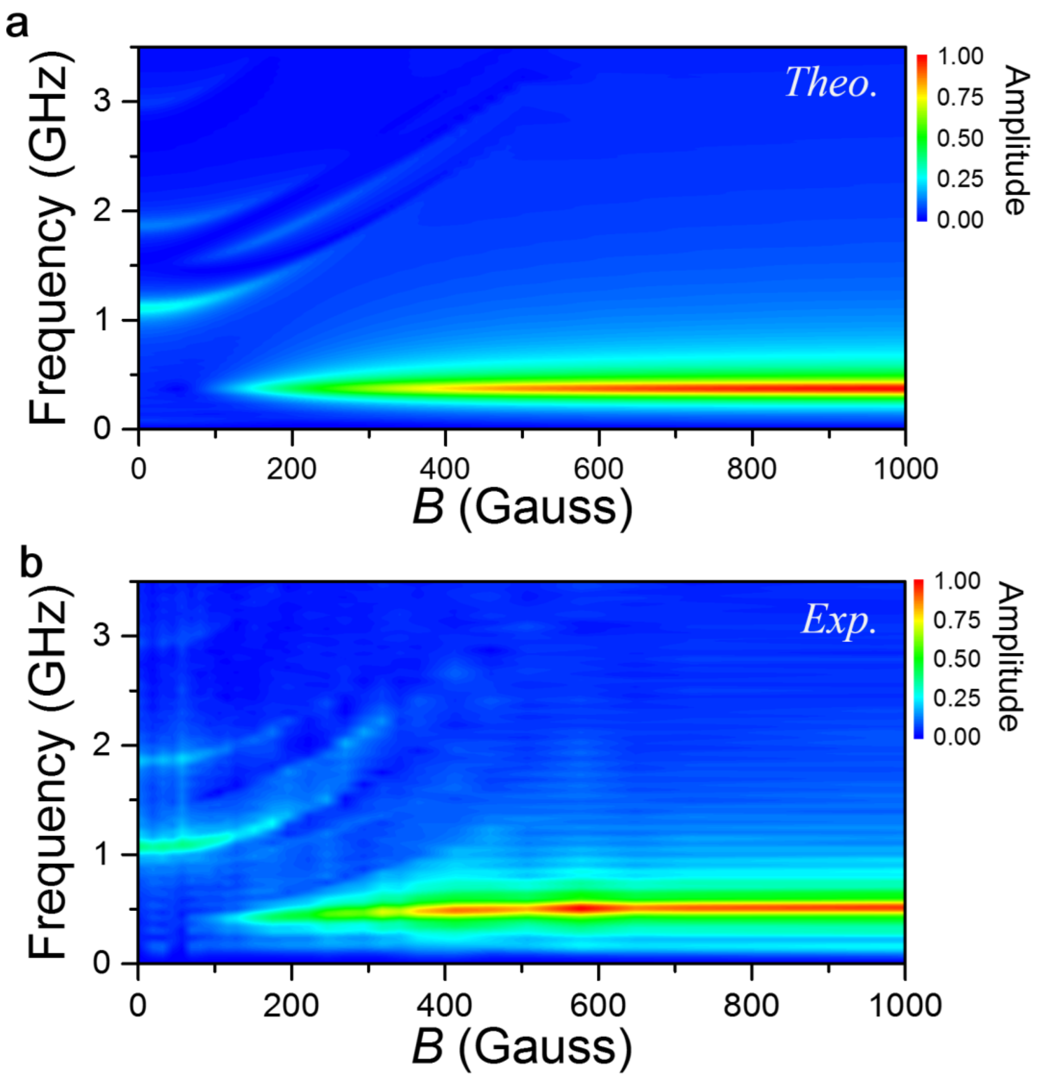

Supplementary Figure 7| Quantum beats with magnetic field along z axis. (a) Theoretical and **(b)** experimental results of quantum beating amplitudes are plotted as functions of the beat frequency and field magnitude.

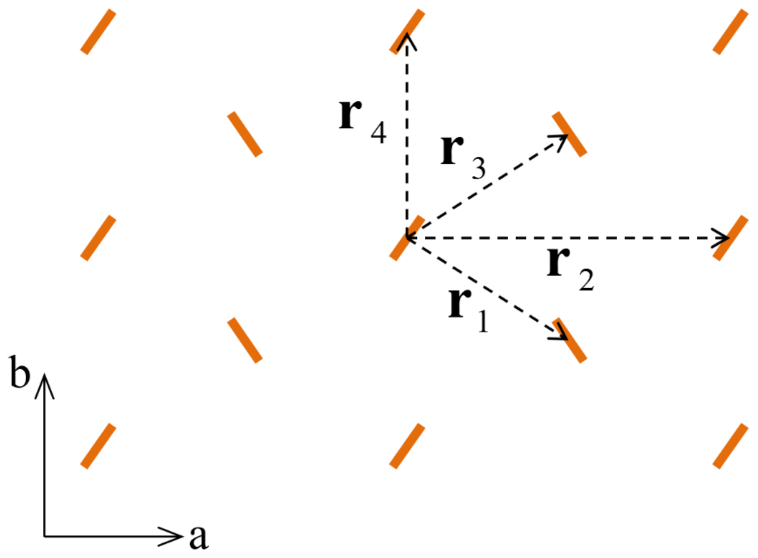

Supplementary Figure 8| Molecular configuration. Schematic diagram of possible configurations for two neighboring molecules in the ab plane of tetracene crystals. The vectors of these configurations in (x,y,z) coordinate system are listed in Supplementary Table 1.

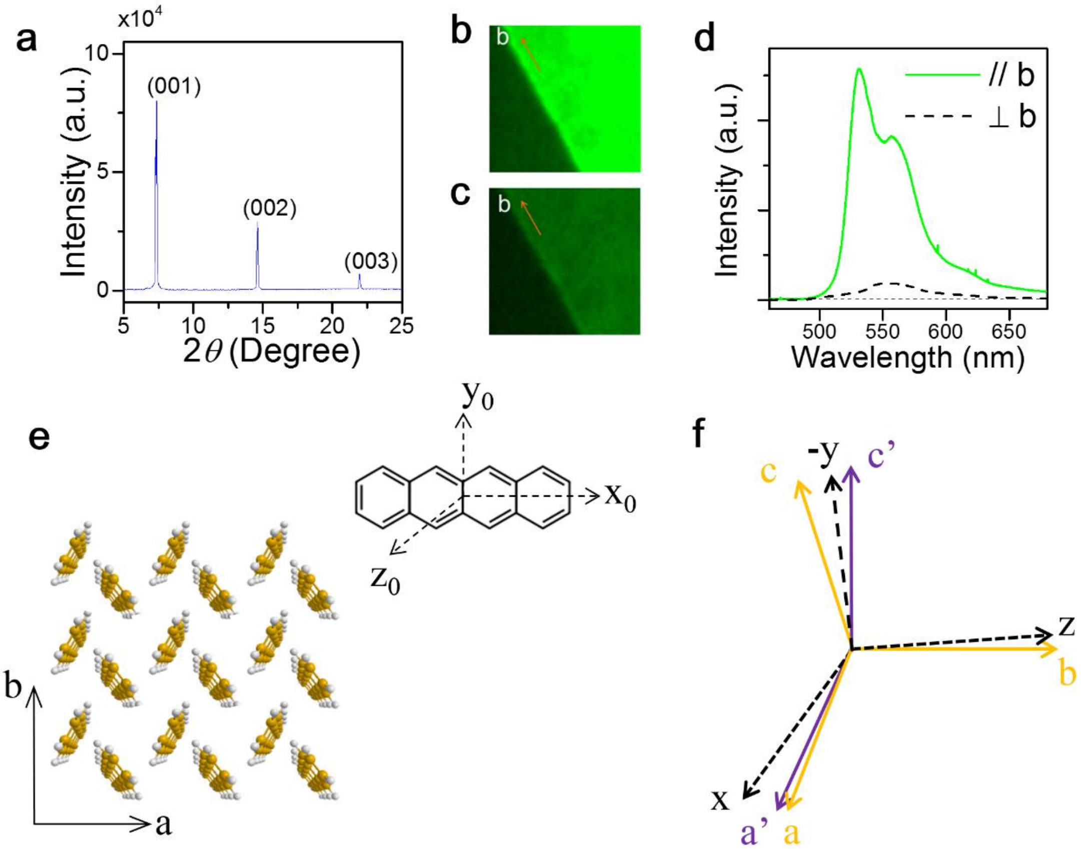

Supplementary Figure 9| Sample characterization and alignment. a, XRD spectrum recorded from a single crystal sample mounted on a glass slide with the largest facet in contact with the substrate. **b** and **c** are photoluminescence microscopy images recorded with polarization parallel and perpendicular to the b axis, respectively. **d,** Photoluminescence spectra recorded with polarization parallel and perpendicular to the b axis, respectively. **e,** Diagram of the in-plane structure of tetracene crystal. Inset shows a tetracene molecule with labelled molecular axes. **f,** An illustration of determining the magnetic axes (x, y, z) with the pre-determined crystal axes (a,b,c).

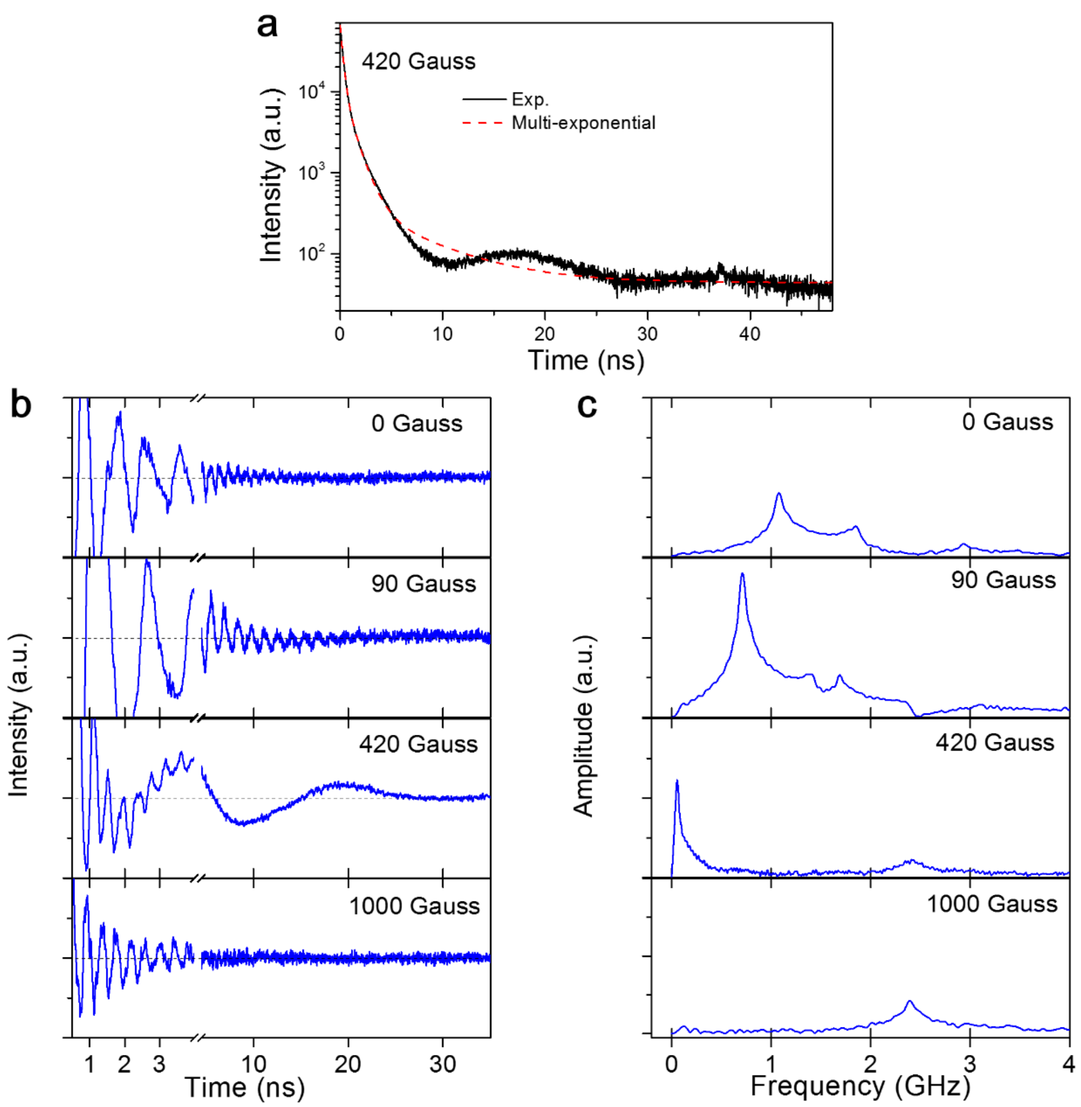

Supplementary Figure 10| Examples of data analysis. The raw data are the same as that used for Figure 2c. **a,** The TRFL trace recorded at 420 Gauss is plotted together with the multi-exponential fitting curve. **b,** The oscillatory components are recorded at different magnitudes of magnetic field. The data are obtained by subtracting the multi-exponential decay curve from raw TRFL traces. **c,** The beat amplitudes are plotted as a function of beat frequency obtained by Fourier transform of the oscillatory components for the same magnetic fields as in **b**. All panels in **b** and **c** are plotted in the same vertical scales, respectively.

Supplementary Table 1. Parameters for four nearest neighboring configuration.

**Configuration**	**Displacement Vector** (n_1,n_2,n_3)	**Distance** (nm)	**Interaction Strength** X (GHz)
$\mathbf{r}_1$	(0.80,0.07,-0.59)	0.48	0.48
$\mathbf{r}_2$	(0.96,0.28,0.03)	0.78	0.11
$\mathbf{r}_3$	(0.72,0.36,0,60)	0.51	0.39
$\mathbf{r}_4$	(-0.03,0.25.0.97)	0.61	0.24

The direction vectors, distances and theoretical values of coupling strength for the four neighboring configurations in tetracene crystals.

Supplementary Table 2. The experimental data of the interaction strength and its equivalent separation distance between the correlated triplet excitons for four possible configurations.

**Configuration**	**Level-crossing resonance**		**Strong-magnetic-field limit**	
	X (GHz)	Distance (nm)	X (GHz)	Distance (nm)
$\mathbf{r}_1$	0.008	1.87	0.008	1.87
$\mathbf{r}_2$	0.007	1.95	0.011	1.68
$\mathbf{r}_3$	0.011	1.68	0.011	1.68
$\mathbf{r}_4$	0.070	0.91	0.008	1.87

Supplementary Table 3. Errors and bounds for various spurious effects.

**Physical mechanisms**	**Resulting signal error**
Uncertainty of beat frequency estimation	$< 1\times10^{-3}$ GHz
Magnitude instability of magnetic field	$< 5\times10^{-5}$ GHz
Spatial heterogeneity of magnetic field	$< 2\times10^{-5}$ GHz
Angle uncertainty of magnetic field	$< 1\times10^{-3}$ GHz
Hyperfine coupling	$< 5\times10^{-4}$ GHz

Supplementary Note 1. Theoretical modelling the quantum beats involving the sublevels of ME state.

In crystalline tetracene, the spin-orbital interaction is negligibly weak. Singlet fission (SF) process is induced by the Hamiltonian operator

$$H = H_{\mathrm{el}} + H_{\mathrm{sp}}\,, \tag{1}$$

where H_{el} and H_{sp} are the spin-free electrostatic part and the spin-dependent part, respectively. The quantum beats are relevant to the manifold sublevels of multiexciton (ME) state that are governed by the spin-dependent Hamiltonian in the form of,

$$H_{\mathrm{sp}}^{\mathrm{TP}} = H_{\mathrm{sp}}^{\alpha} + H_{\mathrm{sp}}^{\beta} + H_{\mathrm{int}}\,. \tag{2}$$

The isolated triplet dipole (e.g., α or β, as represented by “Tri”) can be described by the Hamiltonian [1, 2]

$$H_{\mathrm{sp}}^{\mathrm{Tri}} = g\mu_{\mathrm{B}}\mathbf{B}\cdot\mathbf{S} + D^{*}(S_{z}^{2} - S^{2}/3) + E^{*}(S_{x}^{2} - \text{[illegible]})\,. \tag{3}$$

Here, the first item represents the Zeeman shift due to the applied magnetic field, where $\mathbf{S}$, $\mathbf{B}$, μ_{B}, and g are the spin operator, the external magnetic field, the Bohr magneton, and the Lande g-factor, respectively. The latter two terms give zero-field Hamiltonian with parameters of D^{*} and E^{*} characterized by ESR experiments [3]. The interaction Hamiltonian between the two paired triplets can be written as [4]

$$H_{\mathrm{int}} = X[\mathbf{S}^{\alpha}\cdot\mathbf{S}^{\beta} - 3(\mathbf{S}^{\alpha}\cdot\mathbf{R}/R_{0})(\mathbf{S}^{\beta}\cdot\mathbf{R}/R_{0})]\,, \tag{4}$$

where the interaction strength follows the inverse cubic dependence on the separation distance R_0, i.e.,

$$X = \frac{g^{2}\mu_{\mathrm{B}}^{2}}{R_{0}^{3}}\,. \tag{5}$$

When the interaction is not included, the eigenfunctions and eigenenergies of the nine sublevels of the ME state can be regarded as linear combinations of those for two isolated triplets. Assuming a magnetic field of $\mathbf{B}(B_{\mathrm{x}}, B_{\mathrm{y}}, B_{\mathrm{z}})$, the Hamiltonian for an isolated triplet dipole has the form ($\hbar = 1$):

$$H^{\mathrm{Tri}} = \begin{pmatrix} D^*/3 + g\mu_\mathrm{B}B_\mathrm{z} & \frac{1}{\sqrt{2}}(g\mu_\mathrm{B}B_\mathrm{x} - ig\mu_\mathrm{B}B_\mathrm{y}) & E^* \\ \frac{1}{\sqrt{2}}(g\mu_\mathrm{B}B_\mathrm{x} + ig\mu_\mathrm{B}B_\mathrm{y}) & -2D^*/3 & \frac{1}{\sqrt{2}}(g\mu_\mathrm{B}B_\mathrm{x} - ig\mu_\mathrm{B}B_\mathrm{y}) \\ E^* & \frac{1}{\sqrt{2}}(g\mu_\mathrm{B}B_\mathrm{x} + ig\mu_\mathrm{B}B_\mathrm{y}) & D^*/3 - g\mu_\mathrm{B}B_\mathrm{z} \end{pmatrix} \qquad (6).$$

Here, the spin operators are replaced by Pauli matrices for a system with spin s=1, i.e.,

$$S_\mathrm{x} = \frac{1}{\sqrt{2}}\begin{pmatrix} 0 & 1 & 0 \\ 1 & 0 & 1 \\ 0 & 1 & 0 \end{pmatrix}, S_\mathrm{y} = \frac{1}{\sqrt{2}}\begin{pmatrix} 0 & -i & 0 \\ i & 0 & -i \\ 0 & i & 0 \end{pmatrix}, S_\mathrm{z} = \begin{pmatrix} 1 & 0 & 0 \\ 0 & 0 & 0 \\ 0 & 0 & -1 \end{pmatrix}.$$

The three eigenstates and their eigenvalues for an isolated dipole are obtained by diagonalizing this Hamiltonian (6). We denote the three states (energies) as |x> (E_x), |y> (E_y), and |z> (E_z) for the isolated triplet dipole in analogue to the literature. When the mutual interaction is not considered, the nine ME sublevels of the correlated triplet dipoles can be expressed as a simple combination of states in the form of $\varphi_\mathrm{i}(E_\mathrm{i}) = |\,\alpha\beta > (E_\alpha + E_\beta)$, i.e., |xx> ($2E_\mathrm{x}$), |yy> ($2E_\mathrm{y}$), |zz> ($2E_\mathrm{z}$), |xy> ($E_{\mathrm{x}+}$ E_y), |xz> ($E_{\mathrm{x}+}$ E_z), |yx> ($E_{\mathrm{x}+}$ E_y), |yz> ($E_{\mathrm{y}+}$ E_z), |zx> ($E_{\mathrm{x}+}$ E_z), and |zy> ($E_{\mathrm{y}+}$ E_z).

The magnetic dipolar interaction is sensitive to the displacement vector between the two correlated triplets. The unit vector $\mathbf{R}/R_0$ is defined as (n_1, n_2, n_3) with $n_1^2 + n_2^2 + n_3^2 = 1$. With such interaction, the ME sublevels cannot be expressed as simple combinations of two free triplet dipoles. Generally, one can directly diagonalize the Hamiltonian (2) with the following procedure. The Hamiltonian (2) can be rewritten as

$$H_\mathrm{sp}^\mathrm{TP} = H_\mathrm{zero}^{\alpha+\beta} + H_\mathrm{Zeeman}^{\alpha+\beta} + H_\mathrm{int}. \qquad (7)$$

Here, the first term ($H_\mathrm{zero}^{\alpha+\beta}$) is the zero-field Hamiltonian for two isolated triplets, the second term ($H_\mathrm{Zeeman}^{\alpha+\beta}$) is the field-dependent Hamiltonian for two isolated triplets, and the last term includes the interaction described in (4). The first term can be diagonalized with the interaction-free solution given above, i.e.,

$$H_\mathrm{zero}^{\alpha+\beta} = \sum_{\mathrm{i}=1}^{9} |\,\mathrm{i} > E_\mathrm{i} < \mathrm{i}\,|, \qquad (8)$$

with a basis of nine eigenstates $|\varphi^{\mathrm{i}}_{\mathrm{Zero}}>$. The second Zeeman shift term is then described with a 9×9 matrix Z, i.e.,

$$Z_{\mathrm{ij}} =< \varphi^{\mathrm{i}}_{\mathrm{zero}} \,|\, H^{\alpha+\beta}_{\mathrm{Zeeman}} \,|\, \varphi^{\mathrm{j}}_{\mathrm{zero}} > . \qquad (9)$$

The interaction Hamiltonian part is also represented as a 9×9 matrix I with the same basis, i.e.

$$I_{\mathrm{ij}} =< \varphi^{\mathrm{i}}_{\mathrm{zero}} \,|\, H_{\mathrm{int}} \,|\, \varphi^{\mathrm{j}}_{\mathrm{zero}} > . \qquad (10)$$

The total spin-dependent Hamiltonian is then given in the form of

$$H^{\mathrm{TP}}_{\mathrm{sp}} = \sum_{\mathrm{i=1}}^{9} |\,\mathrm{i} > E_{\mathrm{i}} < \mathrm{i}\,| + \sum_{\mathrm{i=1}}^{9}\sum_{\mathrm{j=1}}^{9} |\,\mathrm{i} > Z_{\mathrm{ij}} < \mathrm{j}\,| + \sum_{\mathrm{i=1}}^{9}\sum_{\mathrm{j=1}}^{9} |\,\mathrm{i} > I_{\mathrm{ij}} < \mathrm{j}\,| . \qquad (11)$$

The energies of the nine ME sublevels ($|\,\phi^{\mathrm{i}}_{\mathrm{TP}} >$) for two coupled triplets can then be calculated by diagonalizing the Hamiltonian (11). For clarity, the sublevels $|\,\phi^{\mathrm{i}}_{\mathrm{TP}} >$ are ranked with index i from 1 to 9 in the sequence of their resonant energies at zero magnetic field. The exact forms of E_{i}, Z_{ij} and I_{ij} are expressed in the end of the Note.

The singlet state (s=0) can be described as

$$|\,\mathrm{S} >= \frac{1}{\sqrt{3}}(|\,0 >|\,0 > - |\,+ >|\,- > - |\,- >|\,+ >) , \qquad (12)$$

where $|\,+ >$, $|\,0 >$, and $|\,- >$ are the eigenfunctions of Pauli matrices. Only the ME sublevels with non-zero mappings to the singlet state (i.e., $\left|\left\langle S_1 \,|\, \phi^{\mathrm{i}}_{\mathrm{TP}} \right\rangle\right|^2 \neq 0$) involve in the exciton fission/fusion processes.

The quantum beats can be simulated with kinetic equations that describe the population of singlet state relevant to the exciton fusion from the ME sublevels. Here, we extend the method derived by Burdett & Bardeen.[1] In principle, to fully describe the evolution involving the singlet state and nine ME sublevels, one should consider the Hamiltonian for the 10-level system as,

$$H = \sum_{\mathrm{n=2}}^{10} |\,\mathrm{n} > \xi_{\mathrm{n}} < \mathrm{n}\,| + \sum_{\mathrm{n=2}}^{10} (|\,\mathrm{n} > M_{\mathrm{n1}} < 1| + |1 > M_{\mathrm{1n}} < \mathrm{n}\,|) , \quad (13)$$

where the energy $|1>$ is the initial singlet state with 0 energy, and M_{1n} represents the transition matrix elements that couple state 1 to the ME sublevels. The dynamical behaviors of the density-matrix elements are governed by the quantum Liouville equation,

$$\frac{\partial \rho}{\partial t} = -\frac{i}{\hbar}[H, \rho] . \quad (14)$$

It is very complicated to fully simulate the dynamics of this 10-level system. To simplify the mathematical procedure, only the ME sublevels involved in the exciton fission/fusion processes will be considered. The mapping to the singlet state (i.e. $\left|\left\langle S_1 | \phi_{\mathrm{TP}}^{\mathrm{i}} \right\rangle\right|^2$) directly reflects the probability of this i^{th} sublevel involving in the exciton fusion/fission processes. We evaluate the mappings for all ME sublevels and include only four sublevels which have the most significant contributions in our simulations of quantum beats with the degenerate sublevels treated as one level having sum of the mappings. The parameters for the simulations are determined with an approach similar to that used in Burdett & Bardeen's work [1].

When no external magnetic field is applied, the eigenvalues for the spin-dependent Hamiltonian of an isolated triplet dipole can be directly calculated with the well-characterized parameters of D^* and E^* [3], i.e.,

$$E_{\mathrm{x}} = D^* / 3 - E^* ,$$

$$E_{\mathrm{y}} = D^* / 3 + E^* ,$$

$$E_{\mathrm{z}} = -2D^* / 3 . \quad (15\text{ a-c})$$

When the interaction is not considered, only three ME sublevels (|xx>, |yy>, and |zz>) have non-zero mappings to the singlet state at zero field case. The energy separations between these levels can well explain the three beat frequencies observed at zero magnetic field [1, 5].

When an external magnetic field is applied along x axis, the term of Zeeman shift in Hamiltonian will lead to significant changes of the ME sublevels. For the interaction-free case, the energies for ME sublevels can be directly calculated as combinations of eigenvalues of Hamiltonian (6). The values are plotted in

Supplementary Fig. 1a, where some ME sublevels are degenerate (i.e., |xy>/|yx>, |xz>/|zx>, and |yz>/|zy>). We calculate the mappings of ME sublevels to the singlet state as shown in Supplementary Fig. 2a. The mappings of ME sublevels (|xy>, |yx>, |xz>, and |zx>) to the singlet state are zero, indicating that these sublevels are not involved in the exciton fusion/fission processes. The quantum beats can then be simulated by considering evolutions of the rest five sublevels. The degenerate sublevels of |yz> and |zy> are taken as a single level to simplify the mathematical procedure. The simulated amplitudes of quantum beating oscillations are plotted as functions of the beat frequency and magnetic field magnitude in Supplementary Fig. 3. The theoretically calculated results can well explain the measured data in the high frequency range (> 0.1 GHz) (Fig. 2c), but the calculation fails to account for the unusual low-frequency beat signal near 420 Gauss.

When the magnetic dipolar interaction is considered, the ME sublevels cannot be regarded as linear combinations of eigenstates for isolated triplets. To calculate the energies of these ME sublevels, we exactly diagonalize the Hamiltonian (11). The displacement vector between the two correlated triplet dipoles is assumed to be along the orientation of the nearest neighbor as suggested in literature [6, 7]. Supplementary Fig. 1b plots the calculated energies of the ME sublevels. These levels of $\left|\phi_{\mathrm{TP}}^{\mathrm{i}}\right\rangle$ are ranked with the sequence of their energies at zero field (Supplementary Fig. 1c). The mappings of these ME sublevels to the singlet state are compared in Supplementary Fig. 2b. The sublevels of 2, 5, & 8 with zero mappings to the singlet state are not involved in the exciton fusion/fission processes. Avoided level-crossing near resonances are clearly observed at ~ 420 Gauss, due to interaction between the triplet dipoles Supplementary Fig. 1b. Since the ME sublevels with non-zero singlet mappings contribute to the exciton fusion/fission processes, the beats relevant to the 3-4 sublevels or 6-7-9 sublevels contribute to the measured low-frequency beat signal as highlighted in Supplementary Fig. 1d. Considering the mappings of ME sublevels to the singlet state, the beat signal related to the 3-4 sublevels is dominant as discussed in the manuscript.

In the above calculation, the eigenstates (eigenenergies) of the basis of nine states ($|\varphi^{\mathrm{i}}_{\mathrm{zero}}>$, i=1…9) for zero-field Hamiltonian $H^{\alpha+\beta}_{\mathrm{zero}}$ are |yy> ($2E_{\mathrm{y}}$), |yz> ($E_{\mathrm{y}+}$ E_{z}), |zy> ($E_{\mathrm{y}+}$ E_{z}), |zz> ($2E_{\mathrm{z}}$), |xy> ($E_{\mathrm{x}+}$ E_{y}), |yx> ($E_{\mathrm{x}+}$ E_{y}), |xz> ($E_{\mathrm{x}+}$ E_{z}), |zx> ($E_{\mathrm{x}+}$ E_{z}), and |xx> ($2E_{\mathrm{x}}$), respectively. The values of E_{x}, E_{y}, E_{z} are the same as in Eq.(15). The matrix form of zero-field Hamiltonian $H^{\alpha+\beta}_{zero}$ reads:

$$\begin{pmatrix} 2E_{\mathrm{y}} & 0 & 0 & 0 & 0 & 0 & 0 & 0 & 0 \\ 0 & E_{\mathrm{y}}+E_{\mathrm{z}} & 0 & 0 & 0 & 0 & 0 & 0 & 0 \\ 0 & 0 & E_{\mathrm{z}}+E_{\mathrm{y}} & 0 & 0 & 0 & 0 & 0 & 0 \\ 0 & 0 & 0 & 2E_{\mathrm{z}} & 0 & 0 & 0 & 0 & 0 \\ 0 & 0 & 0 & 0 & E_{\mathrm{x}}+E_{\mathrm{y}} & 0 & 0 & 0 & 0 \\ 0 & 0 & 0 & 0 & 0 & E_{\mathrm{y}}+E_{\mathrm{x}} & 0 & 0 & 0 \\ 0 & 0 & 0 & 0 & 0 & 0 & E_{\mathrm{x}}+E_{\mathrm{z}} & 0 & 0 \\ 0 & 0 & 0 & 0 & 0 & 0 & 0 & E_{\mathrm{z}}+E_{\mathrm{x}} & 0 \\ 0 & 0 & 0 & 0 & 0 & 0 & 0 & 0 & 2E_{\mathrm{x}} \end{pmatrix}.$$

The matrix form (Z) of Zeeman-shift Hamiltonian ($H^{\alpha+\beta}_{\mathrm{Zeeman}}$) is:

$$\begin{pmatrix} 0 & g\mu_{\mathrm{B}}B_{\mathrm{x}} & g\mu_{\mathrm{B}}B_{\mathrm{x}} & 0 & -g\mu_{\mathrm{B}}B_{\mathrm{z}} & -g\mu_{\mathrm{B}}B_{\mathrm{z}} & 0 & 0 & 0 \\ g\mu_{\mathrm{B}}B_{\mathrm{x}} & 0 & 0 & g\mu_{\mathrm{B}}B_{\mathrm{x}} & 0 & -i\,g\mu_{\mathrm{B}}B_{\mathrm{y}} & -g\mu_{\mathrm{B}}B_{\mathrm{z}} & 0 & 0 \\ g\mu_{\mathrm{B}}B_{\mathrm{x}} & 0 & 0 & g\mu_{\mathrm{B}}B_{\mathrm{x}} & -i\,g\mu_{\mathrm{B}}B_{\mathrm{y}} & 0 & 0 & -g\mu_{\mathrm{B}}B_{\mathrm{z}} & 0 \\ 0 & g\mu_{\mathrm{B}}B_{\mathrm{x}} & g\mu_{\mathrm{B}}B_{\mathrm{x}} & 0 & 0 & 0 & -i\,g\mu_{\mathrm{B}}B_{\mathrm{y}} & -i\,g\mu_{\mathrm{B}}B_{\mathrm{y}} & 0 \\ -g\mu_{\mathrm{B}}B_{\mathrm{z}} & 0 & i\,g\mu_{\mathrm{B}}B_{\mathrm{y}} & 0 & 0 & 0 & g\mu_{\mathrm{B}}B_{\mathrm{x}} & 0 & -g\mu_{\mathrm{B}}B_{\mathrm{z}} \\ -g\mu_{\mathrm{B}}B_{\mathrm{z}} & i\,g\mu_{\mathrm{B}}B_{\mathrm{y}} & 0 & 0 & 0 & 0 & 0 & g\mu_{\mathrm{B}}B_{\mathrm{x}} & -g\mu_{\mathrm{B}}B_{\mathrm{z}} \\ 0 & -g\mu_{\mathrm{B}}B_{\mathrm{z}} & 0 & i\,g\mu_{\mathrm{B}}B_{\mathrm{y}} & g\mu_{\mathrm{B}}B_{\mathrm{x}} & 0 & 0 & 0 & -i\,g\mu_{\mathrm{B}}B_{\mathrm{y}} \\ 0 & 0 & -g\mu_{\mathrm{B}}B_{\mathrm{z}} & i\,g\mu_{\mathrm{B}}B_{\mathrm{y}} & 0 & g\mu_{\mathrm{B}}B_{\mathrm{x}} & 0 & 0 & -i\,g\mu_{\mathrm{B}}B_{\mathrm{y}} \\ 0 & 0 & 0 & 0 & -g\mu_{\mathrm{B}}B_{\mathrm{z}} & -g\mu_{\mathrm{B}}B_{\mathrm{z}} & i\,g\mu_{\mathrm{B}}B_{\mathrm{y}} & i\,g\mu_{\mathrm{B}}B_{\mathrm{y}} & 0 \end{pmatrix}.$$

The matrix form (I) of the interaction Hamiltonian H_{int} is given by:

$$\begin{pmatrix} 0 & 0 & 0 & -X(3n_1^2-1) & 0 & 0 & 3Xn_1n_3 & 3Xn_1n_3 & -X(3n_3^2-1) \\ 0 & 0 & -X(3n_1^2-1) & 0 & 3Xn_1n_3 & 0 & 0 & 3i\,Xn_1n_2 & -3i\,Xn_2n_3 \\ 0 & -X(3n_1^2-1) & 0 & 0 & 0 & 3Xn_1n_3 & 3i\,Xn_1n_2 & 0 & -3i\,Xn_2n_3 \\ -X(3n_1^2-1) & 0 & 0 & 0 & 3i\,Xn_1n_2 & 3i\,Xn_1n_2 & 0 & 0 & X(3n_2^2-1) \\ 0 & 3Xn_1n_3 & 0 & -3i\,Xn_1n_2 & 0 & -X(3n_3^2-1) & 0 & 3i\,Xn_2n_3 & 0 \\ 0 & 0 & 3Xn_1n_3 & -3i\,Xn_1n_2 & -X(3n_3^2-1) & 0 & 3i\,Xn_2n_3 & 0 & 0 \\ 3Xn_1n_3 & 0 & -3i\,Xn_1n_2 & 0 & 0 & -3i\,Xn_2n_3 & 0 & -X(3n_2^2-1) & 0 \\ 3Xn_1n_3 & -3i\,Xn_1n_2 & 0 & 0 & -3i\,Xn_2n_3 & 0 & -X(3n_2^2-1) & 0 & 0 \\ -X(3n_3^2-1) & 3i\,Xn_2n_3 & 3i\,Xn_2n_3 & X(3n_2^2-1) & 0 & 0 & 0 & 0 & 0 \end{pmatrix}.$$

Supplementary Note 2. Angle-dependent measurements near the level crossing resonance.

In time-resolved fluorescence (TRFL) traces, the intensity of delayed fluorescence decays in the time scale of nanoseconds. The damping of the oscillatory components makes a poor visibility for the low-frequency beat signal. As described in the manuscript, we introduce a perturbation by tilting the magnetic field in the xy plane (Fig. 2a) to enhance the visibility as depicted in Supplementary Fig. 4. The interaction between the correlated triplets opens a gap (Supplementary Fig. 4a,b), which is further increased when the field tilting is introduced (Supplementary Fig. 4c). When the tilting angle is small ($\theta < 5^{\circ}$), the perturbation-induced gap size is linearly dependent on θ (Supplementary Fig. 4d), which can be used to interpolate the gap size for zero value of θ (Supplementary Fig. 4e). We note here that the effect of a slight tipping of magnetic field with respect to the xy plane can be neglected.

Besides the above discussed avoided level crossings at ~ 420 Gauss, the calculated energy alignments of ME sublevels in Supplementary Fig. 1b also suggest a crossing of levels 4 & 6 with non-zero singlet mappings at ~ 104 Gauss (Supplementary Fig. 5a). However, the beating amplitude, approximated as the product of the mappings of sublevels 4 & 6, is over one order of magnitude weaker than that of sublevels 3 & 4 at ~ 420 Gauss. Moreover, the magnitude of the beat relevant to the sublevels 4 & 6 is also much weaker than the amplitudes of high-frequency beats related to the sublevels 1 & 9 and 3 & 9 at ~ 104 Gauss (Supplementary Fig. 5b). These results explain the absence of low-frequency beat in the field range near 104 Gauss (Fig. 2c).

Supplementary Note 3. Control experiments with magnetic field applied along z axis

We have also calculated the ME sublevels when a magnetic field is applied along z axis (Supplementary Fig. 6). The obtained energies are plotted in Supplementary Fig. 6, and no level-crossing resonance exists. The energy alignments of ME sublevels are similar for calculation with and without the magnetic dipolar interaction (Supplementary Fig. 6a,c). Without interaction, there are five sublevels including two

degenerated ones with non-zero mappings to the singlet state (Supplementary Fig. 6b); When the interaction is included, the avoided degeneracy between two ME sublevels (i.e., |xy> & |yx> for interaction-free case, Supplementary Fig. 6b) results in one sublevel (# 6) having their total mappings to the singlet state (Supplementary Fig. 6d). These facts lead to very similar simulated results of quantum beat for both interaction-free and interaction-involved models with the procedure described above. Considering these sublevels, we simulated the beating amplitudes as functions of the beat frequency and field magnitude, which can well reproduce the experimental data (Supplementary Fig. 7). This control experiment verifies that the avoided level-crossing resonance is a prerequisite for observing abnormal low-frequency beat in the TRFL traces.

Supplementary Note 4. Measurement at the strong field limit.

When the external magnetic field is sufficiently strong, the first term (i.e., the Zeeman shift) in Hamiltonian (3) is predominant in comparison to the other two terms. In this strong field limit, two ME sublevels dominate the mappings to singlet state (Supplementary Fig. 2b)[8], which exhibits a single-frequency beating (Supplementary Fig. 3 and 7b). The strong-field limit had been solved with a perturbative approach[8]. Here, in order to extract the weak strength of magnetic dipolar interaction, we performed the exact diagonalization of the Hamiltonian (11) for accurate solutions. We considered the case with field applied in the xy plane and changed the field direction with angle Φ in respect to x axis. The interaction will introduce a gap when Φ is tuned to approach the degeneracy of the two sublevels. The strength can be then extracted by analyzing the Φ-dependent beat frequency as discussed in the manuscript.

Supplementary Note 5. The separation distance between the correlated triplet excitons.

For tetracene, highly efficient SF has been observed in crystalline tetracene but not in tetracene solution, suggesting that the intermolecular interactions are essential for the SF process. It is generally believed that SF process creates two triplets in two nearest neighboring molecules when one photo-excited molecule shares its energy

with its neighboring molecule [6, 7]. Supplementary Fig. 8 schematically shows the herringbone structure of molecule alignments in tetracene crystals. There are four possible configurations for two nearest neighbors with different directions as labeled in Supplementary Fig. 8. The parameters for these configurations are listed in Supplementary Table 1. Two molecules aligned with $\mathbf{r}_1$ configuration have the shortest separation distance, which has been frequently employed in modelling SF in crystalline tetracene [6, 7]. Here, the four possible configurations of two neighboring molecules and the strengths of magnetic dipolar interactions for these configurations are considered (Supplementary Table 1).

The effect of magnetic dipolar interaction described by Hamiltonian (4) is sensitive to the interaction strength (X) and the displacement vector ($\mathbf{R}/R_0$) between the two correlated triplets. We fit the experimental data recorded at the cases of level-crossing resonance (~ 420 Gauss, Fig. 3b) and strong-magnetic-field limit with the displacement vectors for all four configurations (Supplementary Table 1). The best fitted parameters of interaction strengths and equivalent distances are listed in Supplementary Table 2. The interaction strength estimated with the experimental data (Supplementary Table 2) is over one-order magnitude weaker than that calculated for two nearest neighboring molecules (Supplementary Table 1). Basically, the equivalent separation distance is 2~4 times larger than the intermolecular distance, which can be explained by the effect of exciton delocalization [9-12] as discussed in the manuscript. These results indicate the exciton size may play an important role for achieving highly-efficient SF in organic materials, which might be the reason of much less inefficient SF (~ 3%) in bis(tetracene) molecules [13] in comparison with that in crystalline tetracene. Moreover, the distance of triplet pair can principally be affected by exciton diffusion. The in-plane diffusion constant for triplet excitons is reported to be at the order of 10^{-3} cm^2 s^{-1} [14], which could increase the triplet separation more than 10 nm within a nanosecond. However, this constant, derived from spatiotemporal characteristics of the delay fluorescence at microsecond scale[14], only represents the dissociated triplets. A certain portion of correlated triplet pairs seems to be proximate

since the spin coherence of germinate triplet pair can be observed at tens of nanoseconds. The effect of exciton diffusion on the coherent behavior of correlated triplet pair requires further in-depth study.

Supplementary Methods

Sample growth and characterization. Tetracene powders were purchased from Sigma-Aldrich (sublimed grade, 99.99%). The single-crystal samples were prepared with the standard method of physical vapor deposition in a quartz tube [15]. The vapor of tetracene was carried by the argon flow (20 sccm) and then deposited onto substrates in the crystal growth zone. Single crystals with thickness of ~1 μm and size up to 5×5 mm^2 were synthesized.

The crystallographic system of tetracene crystal at room temperature is triclinic (a = 0.790 nm, b = 0.603 nm, c = 1.353 nm, α = 100.3°, β = 113.2°, γ = 86.3°)[16]. The crystallographic axes were determined by X-ray diffraction (XRD) and polarization microscopy [14]. The sample was mounted on a glass slide with the largest facet in contact with the substrate. In the XRD spectrum, only (001) peak and its high-order peaks are visible (Supplementary Fig. 9a). No other diffraction peaks can be observed with scanning up to 60° (2θ), suggesting that the crystal has the ab plane oriented in parallel to the largest facet. To determine the orientations of the in-plane axes, polarization microscopy was employed (Supplementary Fig. 9b,c). The fluorescence was routed through a linear polarizer and then measured by a fiber spectrometer (Maya, Ocean Optics). Maximum fluorescence light was recorded when the polarization is parallel to the b axis as discussed previously (Supplementary Fig. 9d) [17].

Magnetic field alignment. For the magnetic-field-dependent experiments, we employed a pre-calibrated magnetic coil driven by a DC power supply. The field direction could be rotated 360° by a step motor. The field magnitude was tuned by the applied current with accuracy better than 0.02 % (equivalent to 0.2 Gauss at the field magnitude of 1000 Gauss).

To align the sample at a desirable direction relative to the magnetic field, the sample was mounted on a multi-axis stage with the rotation/tilt accuracy better than 1 arc minute. Tetracene crystals have molecules aligned in a herringbone pattern in the ab plane (Supplementary Fig. 9e). The magnetic axes of the crystal are different from those of single molecules (Inset, Supplementary Fig. 9e). As suggested in previous

literature, the magnetic axes (x, y, z) of the crystal sample can be determined with the pre-determined crystallographic axes by a transformation matrix as established in the electron spin resonance experiments [3]. Briefly, the new axes of a’ and c’ are defined, respectively, as the in-plane directions normal to b axis and the direction normal to ab plane as depicted in Supplementary Fig. 9f. The orthogonal system of magnetic axes (x, y, z) is transformable with the system of (a’, b, c’) by the following matrix equation [3]

$$\begin{pmatrix} a' \\ b \\ c' \end{pmatrix} = \begin{pmatrix} 0.9634 & 0.2634 & 0\text{[illegible]} \\ -0.0269 & 0.2463 & 0.9\text{[illegible]} \\ 0.2663 & 0.9330 & 0.2\text{[illegible]} \end{pmatrix} \begin{pmatrix} \text{[illegible]} \end{pmatrix} \quad (16).$$

With the operations of rotation and tilting, the magnetic axes can be roughly assigned with an error of better than 0.5 arc degree. The exact directions can then be accurately calibrated by linear interpolations of angle-dependent measurements as discussed in the following section.

Optical setup. The excitation source was a femtosecond Ti:sapphire oscillator (Vitara, Coherent) producing pulses at a wavelength of 800 nm with a temporal duration of ~ 35 fs. The high repetition rate of 80 MHz for the pulses was reduced down to 4 MHz by a pulse picker. The beam was focused onto a BBO crystal to generate frequency-doubled beam (at 400 nm) for TRFL measurements with the technique of time-correlated single-photon counting (PicoHarp 300, PicoQuant) as described elsewhere [18]. The fluorescence was collected and routed to a spectrograph (SP2500, Princeton Instruments). The emission was analyzed by an avalanche photodetector (MPD PDM series, PicoQuant) at the peak wavelength of 530 nm with a temporal resolution of ~ 50 ps. A long-pass optical filter (Di02-R405, Semrock) was employed to eliminate the excitation residual. To avoid the effect of exciton-exciton annihilation, we kept the excitation flux at a low level of ~ 4 nJ cm^{-2} (photon flux ~ 8×10^{9} cm^{-2}).

Data analysis. The quantum beats manifest themselves as oscillations which are entangled with multi-exponential decay components in TRFL traces [1, 5]. To extract the amplitudes and frequencies of quantum beating signals, the multi-exponential decay components were subtracted (Supplementary Fig. 10a). The resultant damped

oscillatory components (Supplementary Fig. 10b) were then analyzed by Fourier transform. Typical examples for analyzing the data plotted in Fig. 2c are shown in Supplementary Fig. 2. At zero field, three peaks at 1.07, 1.84 and 2.95 GHz are observed in the frequency domain which are consistent with results from literature [1]. More frequencies of quantum beats appear when increasing the field up to 90 Gauss. At 420 Gauss, in addition to the above GHz beating components, an anomalous low-frequency beat signal emerges. At 1000 Gauss, a single frequency beat becomes dominant similar to the case of strong-field limit[8]. For the low-frequency beating signal, the frequency has also been evaluated by directly measuring the oscillation period, which gives similar results as that obtained with the method of Fourier transform. The error in estimating the beat frequency is less than 1×10^{-3} GHz.

Since the magnetic dipolar interaction is of small magnitude, we have evaluated the measurement errors and some other possible mechanisms which could affect the accuracy of our measurements. As listed in Supplementary Table 3, we have considered the error bounds relevant to the uncertainties in measuring the beat frequency, the magnitude and angle of magnetic field, as well as the spatial heterogeneity of magnetic field. Moreover, we also estimated the possible effect of the hyperfine interaction. In an isolated tetracene molecule, the hyperfine coupling constants for C-H radicals have been reported to be 1.15, 1.55, and 4.25 Gauss at three different nonequivalent sites of the molecule, respectively[19, 20]. Following the procedures in literature[21, 22], the estimated magnitude of hyperfine coupling for a triplet exciton is less than 2.8 Gauss, corresponding to a deviation of ~ 5×10^{-4} GHz in beating frequency at the high-field limit. In the crystalline sample employed in our study, the effect of hyperfine coupling for triplet exciton is much less efficient since the hyperfine interaction for triplet exciton in organic crystals should be “washed out” as well-established in early literatures [23, 24].

Supplementary References:

1.Burdett JJ & Bardeen CJ. Quantum beats in crystalline tetracene delayed fluorescence due to triplet pair coherences produced by direct singlet fission. *J. Am. Chem. Soc.* **134**, 8597-8607 (2012).

2.Benk H & Sixl H. Theory of 2 coupled triplet-states application to bicarbene structures. *Mol. Phys.* **42**, 779-801 (1981).

3.Yarmus L, Rosentha.J & Chopp M. Epr of triplet excitons in tetracene crystals-spin polarization and role of singlet exciton fission. *Chem. Phys. Lett.* **16**, 477-481 (1972).

4.Wagemans W, *et al.* Spin-spin interactions in organic magnetoresistance probed by angle-dependent measurements. *Phys. Rev. Lett.* **106**, 196802 (2011).

5.Zhang B, *et al.* Nonlinear density dependence of singlet fission rate in tetracene films. *J. Phys. Chem. Lett.* **5**, 3462-3467 (2014).

6.Yost SR, *et al.* A transferable model for singlet-fission kinetics. *Nat. Chem.* **6**, 492-497 (2014).

7.Zimmerman PM, Bell F, Casanova D & Head-Gordon M. Mechanism for singlet fission in pentacene and tetracene: From single exciton to two triplets. *J. Am. Chem. Soc.* **133**, 19944-19952 (2011).

8.Burdett JJ, Piland GB & Bardeen CJ. Magnetic field effects and the role of spin states in singlet fission. *Chem. Phys. Lett.* **585**, 1-10 (2013).

9.Camposeo A, *et al.* Polarized superradiance from delocalized exciton transitions in tetracene single crystals. *Phys. Rev. B* **81**, 033306 (2010).

10.Burdett JJ, Mueller AM, Gosztola D & Bardeen CJ. Excited state dynamics in solid and monomeric tetracene: The roles of superradiance and exciton fission. *J. Chem. Phys.* **133**, 144506 (2010).

11.Voigt M, *et al.* Picosecond time resolved photoluminescence spectroscopy of a tetracene film on highly oriented pyrolytic graphite: Dynamical relaxation, trap emission, and superradiance. *J. Chem. Phys.* **127**, 114705 (2007).

12.Lim SH, Bjorklund TG, Spano FC & Bardeen CJ. Exciton delocalization and superradiance in tetracene thin films and nanoaggregates. *Phys. Rev. Lett.* **92**, 107402 (2004).

13.Mueller AM, Avlasevich YS, Schoeller WW, Muellen K & Bardeen CJ. Exciton fission and fusion in bis(tetracene) molecules with different covalent linker structures. *J. Am. Chem. Soc.* **129**, 14240-14250 (2007).

14.Akselrod GM, *et al.* Visualization of exciton transport in ordered and disordered molecular solids. *Nat. Commun.* **5**, 3646 (2014).

15.Podzorov V. Organic single crystals: Addressing the fundamentals of organic electronics. *MRS Bull.* **38**, 15-27 (2013).

16.Sondermann U, Kutoglu A & Bassler H. X-ray-diffraction study of the phase-transition in crystalline tetracene. *J. Phys. Chem.* **89**, 1735-1741 (1985).

17.Zhang B, *et al.* Polarization-dependent exciton dynamics intetracene single crystals. *J. Chem. Phys.* **141**, 244303 (2014).

18.Xiao J, *et al.* Carrier multiplication in semiconductor nanocrystals detected by energy transfer to organic dye molecules. *Nat. Commun.* **3**, 1170 (2012).

19.Colpa JP & Bolton JR. Hyperfine coupling constants and their dependence on charge densities. *Mol. Phys.* **6**, 273-282 (1963).

20.Lagendijk A, Tromp NFM, Glarsbeek M & van Voorst JDW. Endor in solutions of mononegative ions of aromatic hydrocarbons. *Chem. Phys. Lett.* **6**, 152-154 (1970).

21.Hutchison C & Mangum BW. Paramagnetic resonance absorption in naphthalene in its phosphorescent state. *J. Chem. Phys.* **34**, 908 (1961).

22. Schulten K & Wolynes PG. Semi-classical description of electron-spin motion in radicals including effect of electron hopping. *J. Chem. Phys.* **68**, 3292-3297 (1978).
23. Sternlicht H & McConnell HM. Paramagnetic excitons in molecular crystals. *J. Chem. Phys.* **35**, 1793 (1961).
24. Yarmus L, Rosentha.J & Chopp M. Epr of triplet excitons in tetracene crystals - spin polarization and role of singlet exciton fission. *Chem. Phys. Lett.* **16**, 477 (1972).
